# *MGA* loss-of-function variants cause premature ovarian insufficiency

**DOI:** 10.1172/JCI183758

**Published:** 2024-11-15

**Authors:** Shuyan Tang, Ting Guo, Chengcheng Song, Lingbo Wang, Jun Zhang, Aleksandar Rajkovic, Xiaoqi Lin, Shiling Chen, Yujun Liu, Weidong Tian, Bangguo Wu, Shixuan Wang, Wenwen Wang, Yunhui Lai, Ao Wang, Shuhua Xu, Li Jin, Hanni Ke, Shidou Zhao, Yan Li, Yingying Qin, Feng Zhang, Zi-Jiang Chen

**Affiliations:** 1 Obstetrics and Gynecology Hospital, State Key Laboratory of Genetic Engineering, Institute of Medical Genetics and Genomics, Fudan University, Shanghai, China.; 2 State Key Laboratory of Reproductive Medicine and Offspring Health, Shandong University, Jinan, China.; 3 Shanghai Key Laboratory of Metabolic Remodeling and Health, Institute of Metabolism and Integrative Biology, Fudan University, Shanghai, China.; 4 Center for Reproductive Medicine, Department of Gynecology and Obstetrics, Nanfang Hospital, Southern Medical University, Guangzhou, China.; 5 Department of Pathology, Obstetrics, Gynecology and Reproductive Sciences, University of California San Francisco, San Francisco, California, USA.; 6 Human Phenome Institute, Zhangjiang Fudan International Innovation Center and; 7 School of Life Sciences, Fudan University, Shanghai, China.; 8 Department of Obstetrics and Gynecology, Tongji Hospital, Tongji Medical College, Huazhong University of Science and Technology, Wuhan, China.; 9 Shanghai Key Laboratory of Embryo Original Diseases, Soong Ching Ling Institute of Maternity and Child Health, International Peace Maternity and Child Health Hospital of China Welfare Institute, Shanghai, China.; 10 Shandong Key Laboratory of Reproductive Medicine, Shandong Provincial Hospital Affiliated to Shandong First Medical University, Jinan, China.; 11 Research Unit of Gametogenesis and Health of ART-Offspring, Chinese Academy of Medical Sciences (no. 2021RU001), Jinan, China.; 12 Shanghai Key Laboratory for Assisted Reproduction and Reproductive Genetics, Shanghai, China.

**Keywords:** Genetics, Reproductive biology, Genetic diseases, Monogenic diseases

## Abstract

Although premature ovarian insufficiency (POI), a common cause of female infertility and subfertility, has a well-established hereditary component, the genetic factors currently implicated in POI account for only a limited proportion of cases. Here, using an exome-wide, gene-based case-control analysis in a discovery cohort comprising 1,027 POI cases and 2,733 ethnically matched women controls from China, we found that heterozygous loss-of-function (LoF) variants of MAX dimerization protein (*MGA*) were significantly enriched in the discovery cohort, accounting for 2.6% of POI cases, while no *MGA* LoF variants were found in the matched control females. Further exome screening was conducted in 4 additional POI cohorts (2 from China and 2 from the United States) for replication studies, and we identified heterozygous *MGA* LoF variants in 1.0%, 1.4%, 1.0%, and 1.0% of POI cases, respectively. Overall, a total of 37 distinct heterozygous *MGA* LoF variants were discovered in 38 POI cases, accounting for approximately 2.0% of the total 1,910 POI cases analyzed in this study. Accordingly, *Mga^+/−^* female mice were subfertile, exhibiting shorter reproductive lifespan and decreased follicle number compared with WT, mimicking the observed phenotype in humans. Our findings highlight the essential role of MGA deficiency for impaired female reproductive ability.

## Abstract 2

*MGA* could explain 1.0%-2.6% POI cases, representing as the TOP 1 among the genes whose coding variants can cause POI.

## Introduction

Premature ovarian insufficiency (POI) is a disorder characterized by impaired ovarian function, reduced fertility, and premature menopause (1). POI affects 1%−3.5% of women (2–4). Previous studies have identified significant correlations in menopausal age between mothers and their daughters (5) and high heritability (44%−65%) of menopausal age estimated using mother-daughter pairs, illustrating the strong genetic basis of POI. Additionally, 14%−31% of POI cases have a family history, among whom 30%−55% had an affected mother (6, 7). Despite advances in the understanding through animal models and molecular genetics, only a fraction of the known genetic causes of POI have been identified, accounting for approximately 30%−40% of cases (8–13). This discrepancy between the apparently high heritability of ovarian aging and the limited contribution of known genetic factors to total POI cases strongly suggests the presence of missing heritability and the existence of potential genetic factors yet to be uncovered (14).

Furthermore, recent studies have reported that coding variants in any single known POI causal gene account for only approximately 1% or less of total POI cases (8–11). Consequently, this extensive genetic heterozygosity of POI presents a formidable obstacle in the discovery of novel causal genes, particularly in sporadic cases with limited sample sizes. In addition, these studies mainly employed strategies for identifying POI genes among candidates with supported annotations for ovarian functions and therefore may have overlooked POI causative genes beyond the scope of screening. This relative dearth of pathogenic genes in extensive screening suggests that a nontargeted strategy may be necessary to identify novel genes for POI.

An “anonymous” gene-based burden analysis, which requires no prior knowledge of gene functional annotation, has been previously established and subsequently proven effective for identifying causal genes in a variety of diseases with characteristically high allelic heterogeneity (15–17). Here, to uncover core pathogenic POI genes among exome-wide human coding genes, we employed this approach in the currently largest-scale sequencing dataset of POI cases and ancestry-matched women controls.

## Results

### MGA loss-of-function variants are significantly enriched in POI, but rare in human populations.

**Figure 1:**
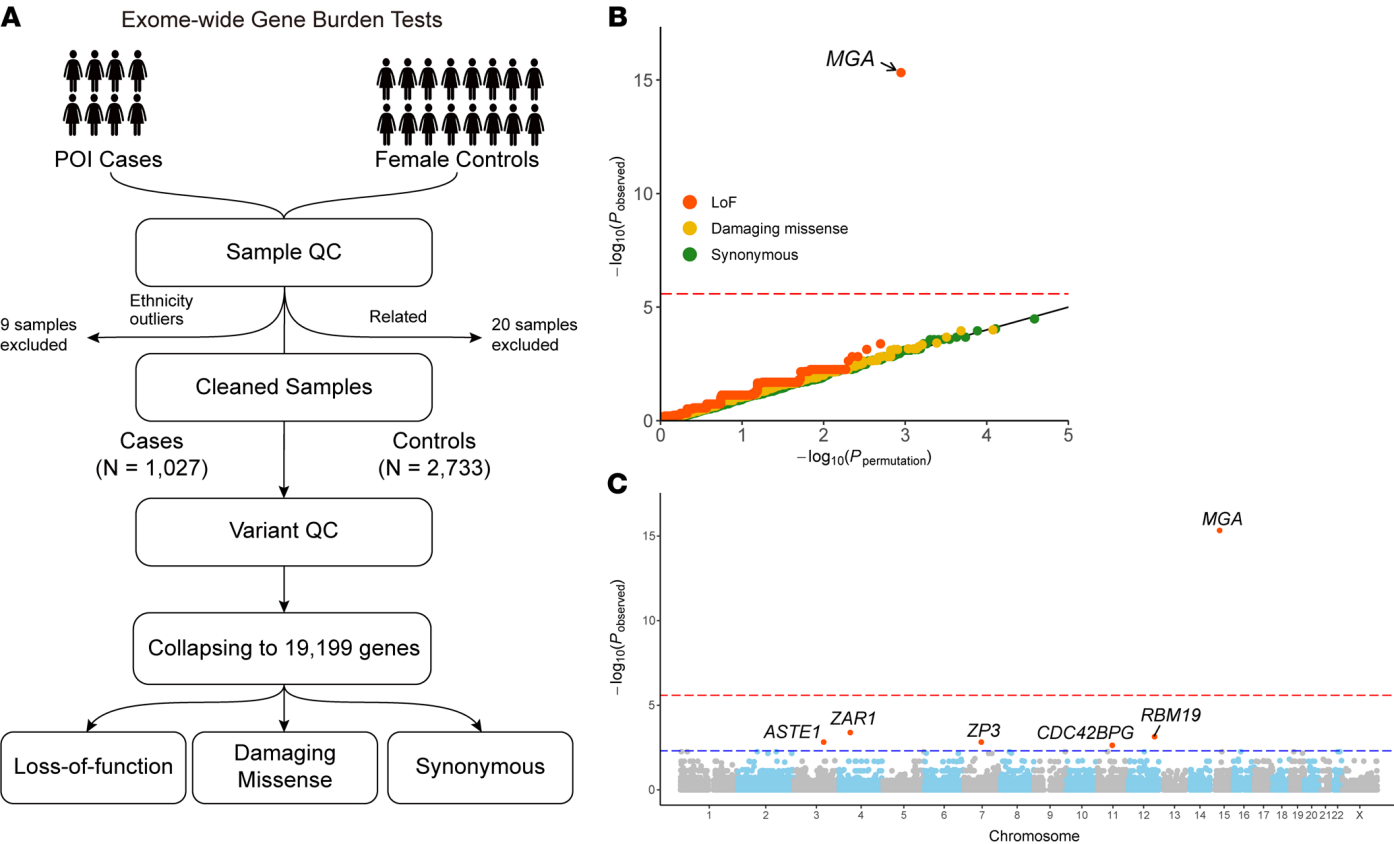
Identification of *MGA* as a POI-associated gene in the discovery cohort. (**A**) Genetic analysis pipeline of exome-wide gene burden tests using WES data in the discovery POI cohort and matched female controls. (**B**) Gene-based Q-Q plot of *P* values in the discovery stage based on 2-sided Fisher’s exact tests. Expected *P* values are obtained from 1,000 permutations. The burden of variants adhering to 3 variant types, namely LoF, damaging missense, and synonymous, was tested for each of the 19,199 genes. Points on the plot are color coded according to the variant types indicated in the legend. The inflation factors (λ) for the 3 variant types are 1.08, 1.03, and 1.00, respectively. The red dashed line represents the Bonferroni’s correction threshold of 2.6 × 10^–6^ (0.05/19,199) for each variant type. (**C**) Manhattan plot illustrating the association between genes with LoF variants and POI from the discovery cohort. The –log_10_(observed *P* values) are plotted against the genetic position for each analyzed gene. The red dashed line represents the Bonferroni’s correction threshold, and the blue dashed line indicates a suggestive significance threshold of 0.005. The genes achieving the suggestive significance threshold are labeled.

Our primary association analysis of the whole exome sequencing (WES) data consisted of 1,027 unrelated POI cases (cohort 1) and 2,733 ethnically matched control women after sample quality control (QC) (Figure 1A, Supplemental Figures 1 and 2, and Supplemental Table 1; supplemental material available online with this article; https://doi.org/10.1172/JCI183758DS1). After variant QC and filtering out variants with low quality and a minor allele frequency (MAF) > 0.1%, a total of 19,199 human coding genes were found to harbor at least 1 loss-of-function (LoF), damaging missense, or synonymous variant. These 19,199 genes were compared for the burdens for these 3 variants types using 2-sided Fisher’s exact tests. Importantly, gene-burden analyses for all 3 models demonstrated negligible inflation (λ = 1.08, 1.03, and 1.00), indicating a high level of concordance between the cases and controls (Figure 1B). Promisingly, among all the burden tests performed, the LoF model revealed a significant association of MAX dimerization protein (*MGA*), yielding the highest association with a *P* value of 4.7 × 10^–16^. Notably, *MGA* was the only gene that achieved exome-wide significance under Bonferroni’s correction (<0.05/19,199), followed by the previously reported POI causative gene *ZAR1* (Figure 1C) (11, 18). A total of 26 *MGA* LoF variants in 27 (2.6%) cases were identified in the discovery cohort (Table 1), while no *MGA* LoF variants were identified in matched control women. This finding highlights the potential role of *MGA* in the development of POI.

We further investigate the prevalence of *MGA* LoF variants in general populations using multiple public genomic datasets. To ensure the consistency of our analyses, variants were annotated using the same canonical transcript of *MGA* as that used for the above association analyses (NM_001164273.2). Variants were excluded if they only introduced in-frame alterations as determined by mini-gene assay or if they were found to be in cis within the same individual and introduced in-frame alterations (Supplemental Figure 3). This analysis revealed that the frequencies of individuals harboring *MGA* LoF variants were 0.0% in the HuaBiao project (19) and the NyuWa Genome resource (20), 0.0094% in the ChinaMAP (21), 0.0014% in the Mexico City Prospective Study of the Regeneron Genetics Center (22), 0.0093% in the BRAVO (TOPMed release 10) (23), and 0.014% in the gnomAD, version 4.1.0, databases (24) (Table 1). Using the combined data of the above human genomic databases, we estimated an accumulated prevalence of 0.013% (95% CI, 0.011% to 0.016%) for *MGA* LoF variants in general populations, notably lower than that in POI case cohorts (Table 1). Additionally, based on the data from gnomAD, *MGA* had a pLI (the probability of being LoF intolerance) score of 1.0, indicating that *MGA* is highly likely to be a haploinsufficient gene, i.e., the loss of a single copy of the gene is not tolerated. Overall, these findings in human populations suggested that heterozygous LoF variants in *MGA* are deleterious and subject to high natural selective pressure.

### Replication studies in multiple POI cohorts commonly identified MGA LoF variants.

To further examine the involvement of *MGA* LoF variants in more POI cases, additional POI cohorts from multiple centers of other regions were investigated. Two POI cases (1.0%) in cohort 2 (Guangdong, China) and 7 POI cases (1.4%) in cohort 3 (Hubei, China) harbored heterozygous LoF variants in *MGA* (Table 1), whereas none of the 606 individuals from Hubei or 1,304 individuals from Guangdong had *MGA* LoF variants according to PGG.HAN2 (25), a database integrating sequencing data of individuals with Han Chinese ancestry.

We also investigated the prevalence of *MGA* LoF variants in non–East Asian POI cases. Two POI cohorts recruited at different centers in the US were enrolled in WES analysis to screen *MGA* LoF variants. In the Pittsburgh cohort with 104 POI cases (cohort 4), a heterozygous frameshift deletion c.1673del (p.Asp558Alafs*42) of *MGA* was identified in 1 case who self-identified as White. In the NIH cohort (cohort 5), one of 98 nonsyndromic POI cases was identified as harboring a heterozygous nonsense variant, c.350C>G (p.Ser117*) of *MGA*. *MGA* LoF variants accounted for approximately 1.0% of POI cases in both cohorts from the US (Table 1).

**Figure 2:**
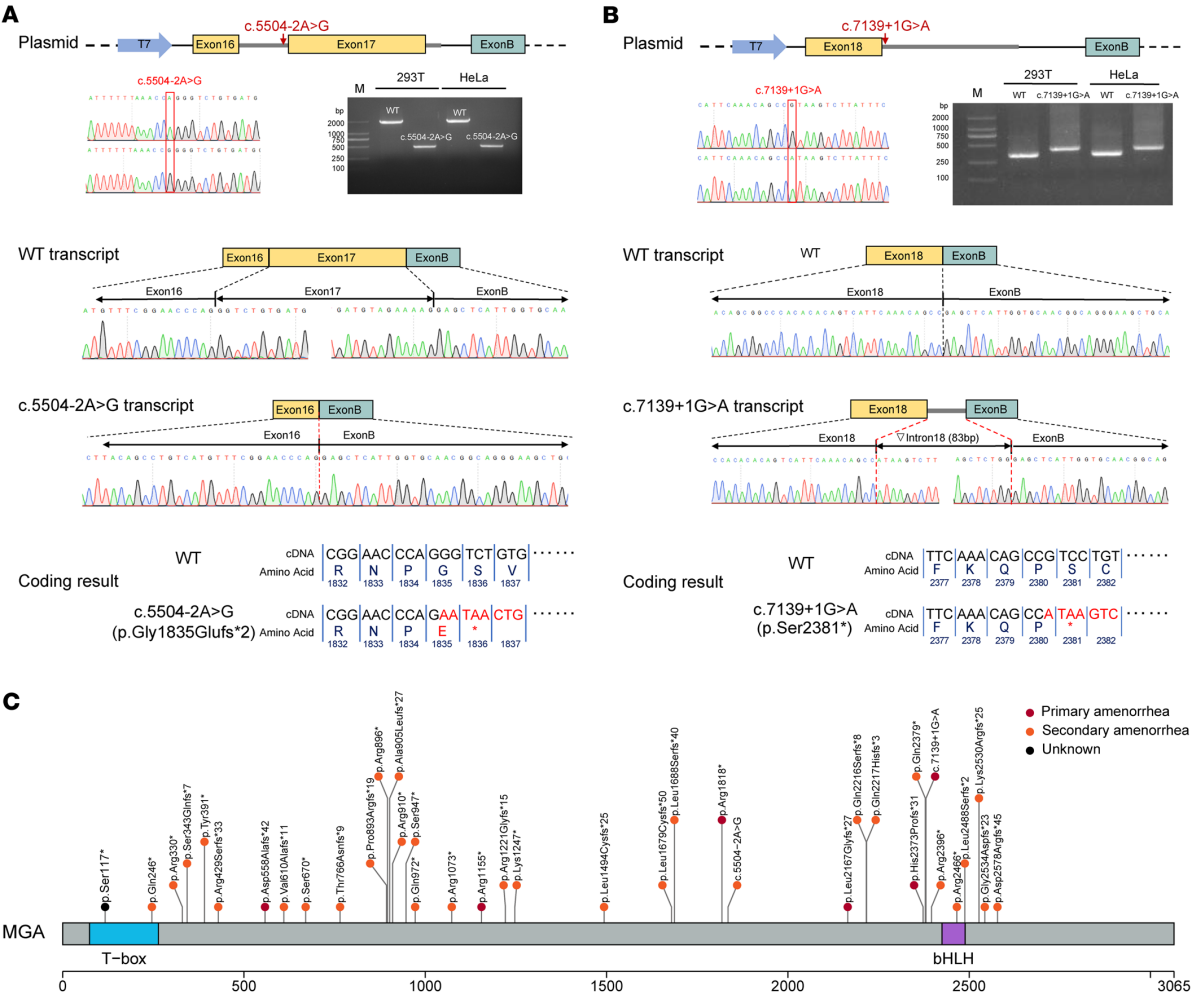
Validation and schematic representation of LoF variants identified in patients with POI. (**A** and **B**) Splicing results of the *MGA* c.5504-2A>G (**A**) and c.7139+1G>A (**B**) variants by mini-gene assays. Sanger sequencing results of the recombinant vectors are shown. Electrophoresis results showing the transcript PCR products obtained from both 293T and HeLa cell lines. The transcript of the c.5504-2A variant results in skipping of exon 17 and introducing a new premature termination in exon 18. The transcript of the c.7139+1G>A variant retained an 83 bp fragment of intron 18 and results in the introduction of a new premature termination codon. Both the c.5504-2A>C and c.7139+1G>A variants had shorter products compared with WT. (**C**) Schematic representation of the MGA protein and the LoF variants identified in patients with POI. The depicted domains include the T-box and basic helix-loop-helix (bHLH) domains of MGA.

The variants identified in POI cases of cohorts 1, 2, and 3 were confirmed by Sanger sequencing (Supplemental Figure 4). As for the variants identified in cohorts 4 and 5, their confirmation involved ensuring adequate coverage of over 100 reads and conducting a manual review of the bam files. The 2 splice-site variants identified in cases were validated by mini-gene assays that both affected the splicing process, resulting in protein truncation (Figure 2, A and B).

Finally, 37 *MGA* LoF variants were identified in 38 POI cases. Among these variants, only 5 were observed in gnomAD or BRAVO, and they had exceedingly rare frequencies (<0.00001); the remaining variants were absent from all the public population databases (Supplemental Table 2). Furthermore, all of them induced premature termination codons before the 3′ most exon and the 3′ most 50 bp of the penultimate exon of the gene and lost at least 10% C-terminal amino acids of the protein (Supplemental Table 2 and Figure 2C). Therefore, they all are predicted to induce nonsense mediated decay and met the criteria of a pathogenic criterion (PVS1) (26). In summary, *MGA* LoF variants cumulatively contributed to 2.0% of a total of 1,910 POI cases analyzed in this study, hundreds of times higher than that in the general population (Table 1).

### Clinical characteristics and familiar inheritance in patients with MGA LoF variants.

Among 38 POI cases identified with *MGA* LoF variants, 37 had normal CGG-repeat expansions (<55) in the well-known POI causal locus, *FMR1*, while the *FMR1* information of the case from cohort 5 was unavailable. The majority (31/37) of these POI cases experienced secondary amenorrhea, and their age of amenorrhea ranged from 13 to 33 years. Detailed clinical characteristics are shown in Supplemental Table 3. No obvious difference was observed in the location distribution of *MGA* variants between cases with primary versus secondary amenorrhea (Figure 2C).

**Figure 3:**
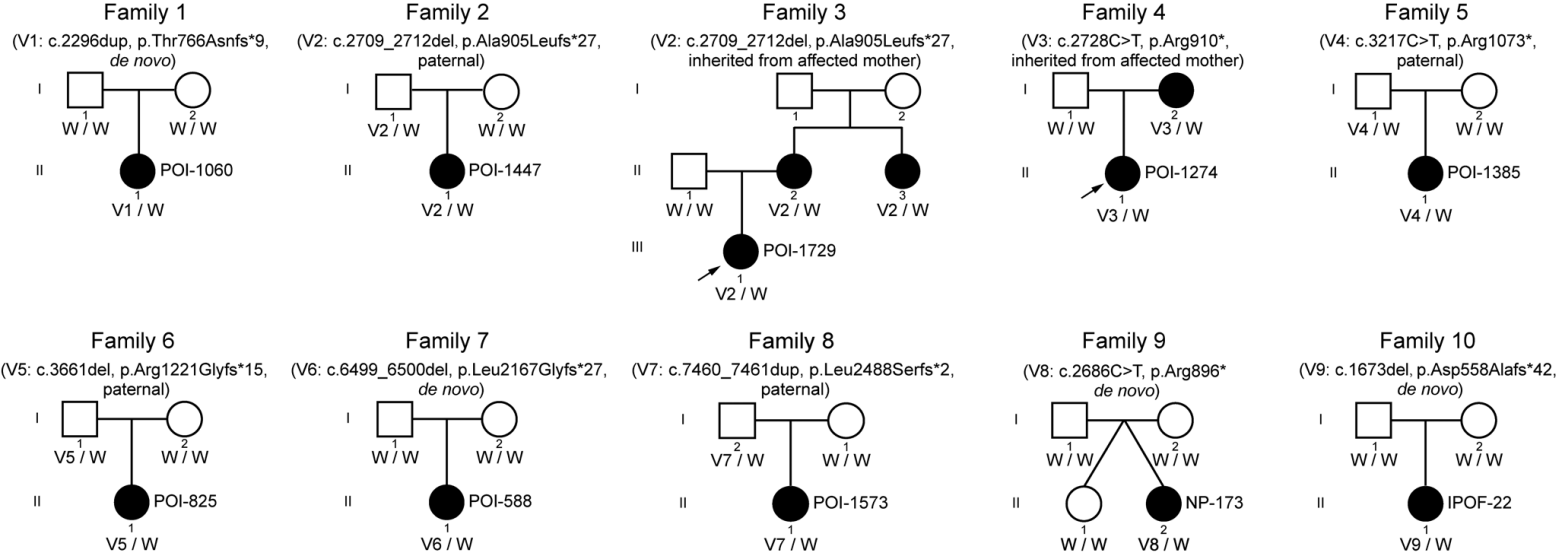
Variant inheritance in 10 independent pedigrees with heterozygous *MGA* LoF variants. Pedigrees of 10 unrelated families with individuals harboring heterozygous LoF variants in *MGA*. The arrows in families 3 and 4 indicate the probands, i.e., the individuals through whom the genetic variants were initially identified. The genotypes of the individuals are indicated below the symbols, with W representing the WT allele.

We next investigated the segregation characteristics of *MGA* LoF variants with POI phenotype. Sanger sequencing and pedigree analysis were performed in 10 pedigrees in which DNA samples of family members were available (Figure 3). Among these pedigrees, 4 variants were found to arise de novo (families 1, 7, 9, and 10) and 4 variants were paternally inherited (families 2, 5, 6, and 8). Interestingly, the *MGA* LoF variant from family 9 was identified as de novo in the proband, while her fraternal twin remained WT and had normal menstruation thus far. The variant identified in POI-1274 was inherited from her mother (family 4, I-2), who also experienced early menopause at age 30 (Supplemental Table 4). Notably, the *MGA* LoF variant identified in POI-1729 was inherited from her mother (family 3, II-2) having menopause at age of 31, and was also carried in her aunt (family 3, II-3), having menopause at the age of 35 (Supplemental Table 4). All of these female family members (including the 2 mothers and 1 aunt) with *MGA* LoF variants experienced idiopathic secondary amenorrhea before the age of 40, indicating early ovarian depletion. These pedigrees provided evidence supporting that *MGA* LoF variants cosegregated with POI in women.

### Heterozygous Mga LoF mutant mice exhibited female subfertility and accelerated ovarian depletion.

**Figure 4:**
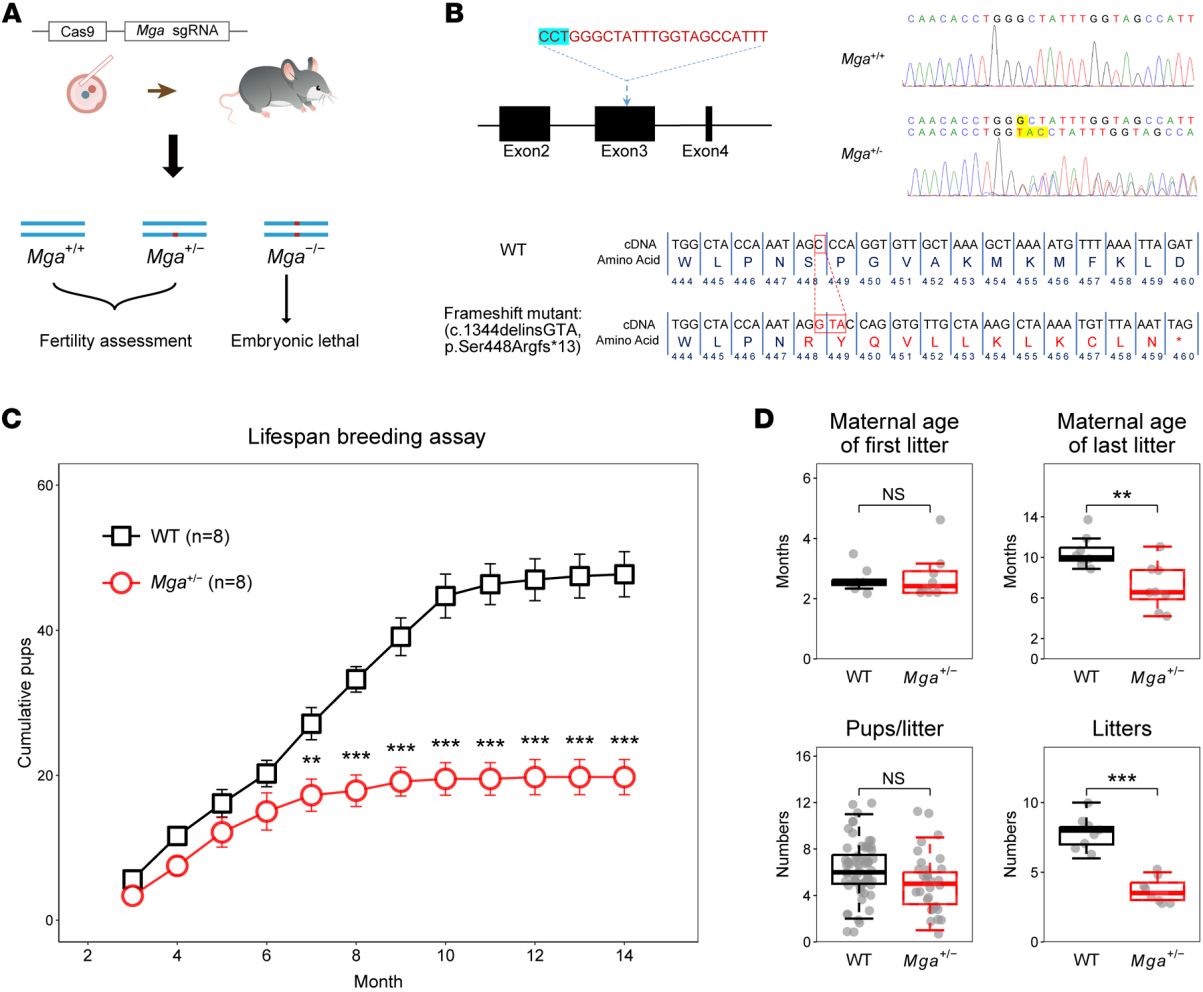
Subfertility of *Mga^+/−^* female mice. (**A**) Schematic illustration of the targeting strategy used to generate *Mga*-mutated mice using CRISPR/Cas9 technology. (**B**) The sgRNA was designed to target exon 3 of mouse *Mga*, and the protospacer adjacent motif is indicated by the blue highlight. Sanger sequencing results of the WT and heterozygous *Mga* mutants showed 1 bp deletion plus 3 bp insertion (c.1344delinsGTA) was introduced in mouse *Mga*, resulting in a frameshift mutation (p.Ser448Argfs*13) of MGA. The mutated nucleotides and amino acids are highlighted in red. The termination codon is indicated by an asterisk. (**C**) Cumulative number of pups produced per female mouse through continuous breeding. Both WT (*n* = 8) females and *Mga^+/−^* (*n* = 8) females were mated with WT male mice. Asterisks indicate statistically significant differences between the 2 groups. (**D**) Statistical summary of breeding/reproductive features of *Mga^+/−^* and WT female mice, including maternal age at first litter, maternal age at last litter, average number of pups per litter, and average number of litters. Central line of boxplots denotes median, box marks interquartile range and whiskers ×1.5 interquartile range. Statistical significances of **C** and **D** were determined using a 2-sided unpaired *t* test. **P < 0.01; ***P <0.001.

In order to investigate the potentially causal relationship between *MGA* LoF variants and POI, we induced a frameshift mutation in *Mga* in C57BL/6 mice (Figure 4, A and B). Homozygous *Mga*-mutated (i.e., *Mga^−/−^*) mice were embryonic lethal, which is consistent with previous studies (27, 28). Importantly, no *MGA* LoF homozygotes were detected in either human data from this study or in the various human genome databases, further supporting the likelihood that homozygous LoF mutations in *Mga*/*MGA* are incompatible with survival in both mice and humans.

To access the reproductive capacity of heterozygous *Mga*-mutated (*Mga^+/−^*) female mice, breeding assays were conducted for over 1 year, involving the mating of *Mga^+/−^* female mice and their littermate WT females with WT male mice. The breeding result showed that heterozygous *Mga*-mutated (*Mga^+/−^*) female mice had a lower cumulative number of pups compared with their WT female littermates (Figure 4C) and ceased giving birth at an average of 6.3 months, over 4 months earlier than WT females (Figure 4D). Furthermore, maternal age at first litter and mean number of pups per litter were not significantly different between the 2 genotypes (Figure 4D), indicating that *Mga^+/−^* females had an overall shorter reproductive lifespan compared with WT female littermates. This reproductive phenotype of *Mga^+/−^* female mice resembled that of POI in *MGA*-associated human cases who experienced early menopause but still retained fertility in some cases (e.g., POI-1565, POI-956, the mother of POI-1729 in family 3, and the mother of POI-1274 in family 4). Notably, *Mga^+/−^* male mice showed no obvious abnormalities in sperm characteristics or fertility compared with WT controls (Supplemental Figure 5).

**Figure 5:**
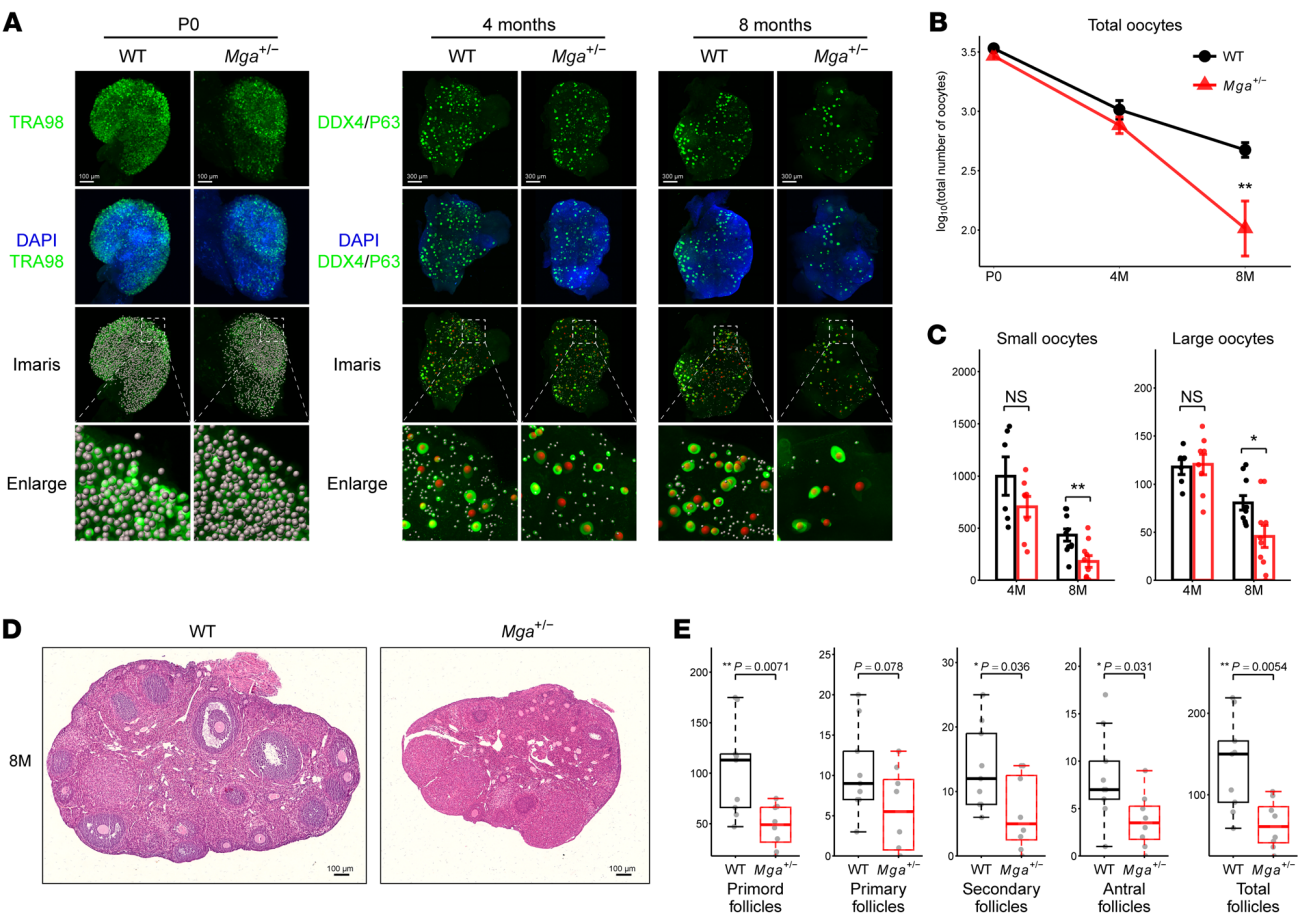
Accelerated depletion of ovarian reserve in *Mga^+/−^* female mice. (**A**) Representative images of reconstructed 3D ovaries. Mouse oocytes collected at P0 were stained with TRA98; oocytes collected at 4M and 8M were costained with DDX4 and P63 antibodies. Data analysis was conducted by spot transformation using Imaris software. Spots representing oocytes are colored based on size. Small oocytes with small diameter between 14 and 40 μm, which represent quiescent oocytes in primordial follicles, are indicated as gray spots. Large oocytes with diameter greater than 40 μm, representing growing oocytes in secondary and antral follicles, are indicated as red spots. The scale bars indicate 100 µm at P0 and 300 µm at both the 4M and 8M time points. The enlarged views are magnified to 5 times the original scale. (**B**) The number of total oocytes per ovary at P0, 4M, and 8M, respectively. Significantly fewer total oocytes were observable in *Mga^+/−^* females compared with WT females at 8M. (**C**) Statistical summary of small and large oocytes per ovary in 4M and 8M between *Mga^+/−^* and WT females. Data in **B** and **C** show means ± SEM for at least *n* = 6 mice per genotype for each time point and can be found in the Supporting Data Values file. (**D**) H&E staining of ovarian sections of 8M mice. (**E**) Box plot showing statistical summary of primordial, primary, secondary, and antral follicles per ovary in WT (*n* = 9) and *Mga^+/−^* (*n* = 8) mice age 8M. Central line of box plots denotes median; box marks interquartile range and whiskers 1.5× interquartile range. Statistical significances of **B**, **C**, and **E** were determined using a 2-sided unpaired *t* test. *P < 0.05; **P < 0.01.

To further investigate the impacts of heterozygous *Mga* mutant on ovarian reserve, we compared the number of oocytes between WT and *Mga^+/−^* female mice at different ages using fluorescence immunostaining for the oocyte markers and 3D ovarian images (Figure 5A) (29). The number of total oocytes, small oocytes (indicating quiescent oocytes in primordial follicles), and large oocytes (indicating growing oocytes in secondary and antral follicles) all showed a significant decrease at 8 months of age in *Mga^+/−^* females compared with WT (Figure 5, B and C). Histological examination of ovarian sections by H&E staining also revealed significant declines in primordial and growing follicles in *Mga^+/−^* mice compared with WT female littermates (Figure 5, D and E). These findings suggested that follicle depletion is accelerated in *Mga^+/−^* females in comparison with WT females, thus supporting the genetic involvement of *Mga* in maintaining ovarian reserve.

### Mga^+/−^ ovaries undergo aberrant transcriptional expression.

Analysis of published transcriptomic data from ovarian tissues indicate that *MGA*/*Mga* is highly expressed in germ cells and granulosa cells (GCs) from early embryonic through adult developmental stages in both humans and mice (Supplemental Figure 6) (30–33), suggesting its potential role in ovaries. *MGA* encodes a MAX dimerization protein and serves as the scaffold component of polycomb repressive complex 1.6 (PRC1.6, a regulatory factor), which is a transcriptional repressor and participates in suppressing germ cell–related gene expression in stem cells and germ cells (34).

**Figure 6:**
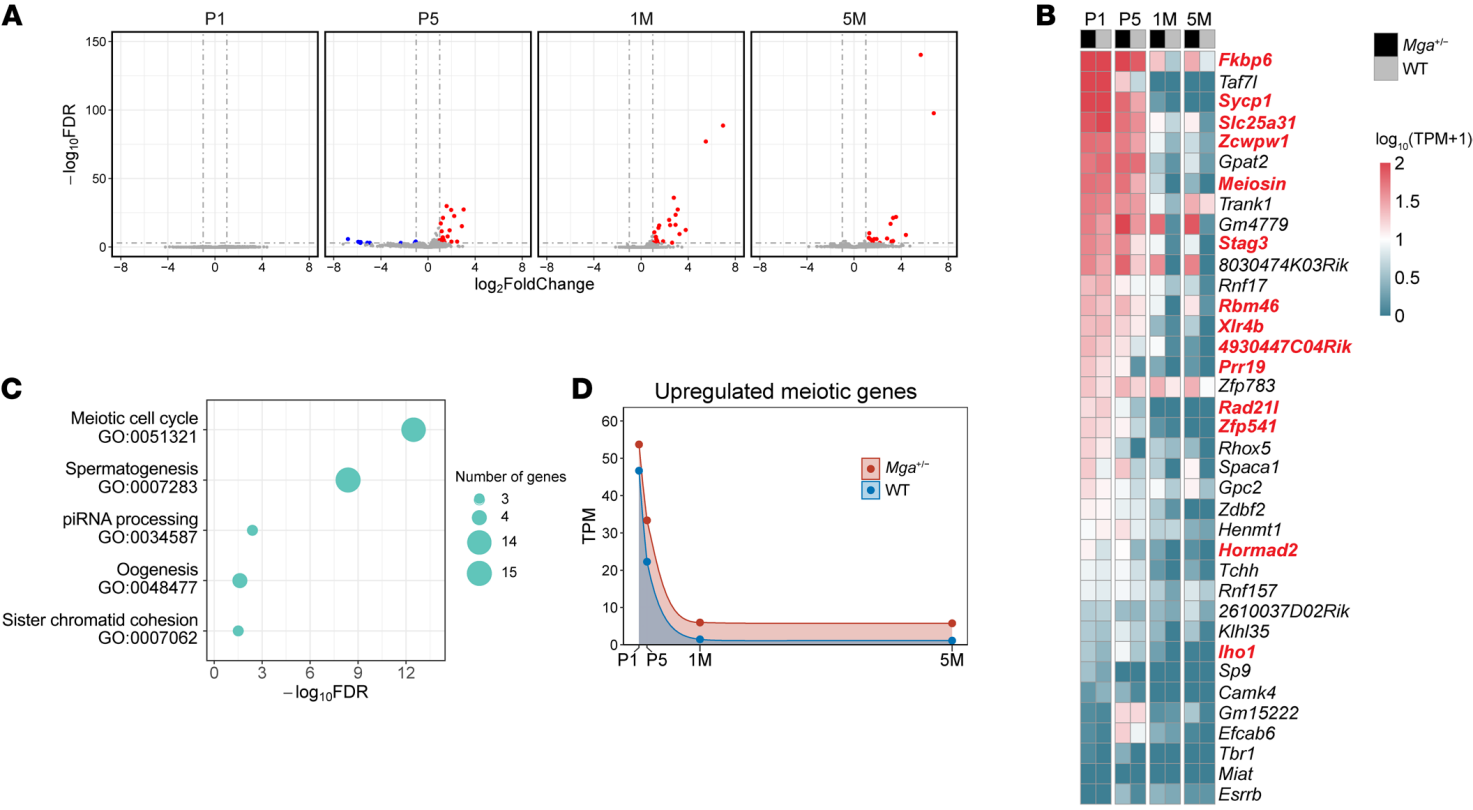
MGA suppresses the transcript expression of meiotic genes in ovary. (**A**) Volcano plots of the gene expression profile in the ovaries between WT and *Mga^+/−^* female mice aged P1, P5, 1M, and 5M, respectively. Bulk RNA-Seq was conducted on at least 3 ovaries of each genotype at each time point. Upregulated differential expressed genes with log_2_FC > 1 and FDR < 0.001 are denoted in red, while downregulated DEGs with log_2_FC < –1 and FDR < 0.001 are denoted in blue. (**B**) Heatmaps showing expressions of the total of 37 upregulated DEGs, 14 of which are meiotic genes and are denoted in bold. (**C**) GO enrichment analyses of 37 upregulated DEGs. (**D**) Expressions of the 14 DEGs related to meiosis in the ovaries of female mice aged P1, P5, 1M, and 5M. Points indicate means.

To investigate the molecular changes in *Mga^+/−^* female mice, we conducted bulk RNA-Seq on ovaries collected at different developmental stages, including P1, P5, 1 month (1M), and 5M of age. We identified 0, 35, 21, and 17 differential expression genes (DEGs) at these respective time points (Figure 6A). Notably, the majority of DEGs were upregulated in *Mga^+/−^* females (Figure 6, A and B), consistent with the known repressive regulatory function of MGA. Gene Ontology (GO) analysis of the 37 upregulated DEGs in *Mga^+/−^* females showed enrichment for processes related to meiosis (Figure 6C and Supplemental Table 5). Interestingly, at P1, both *Mga^+/−^* and WT ovaries exhibited high expression levels of these meiosis-related genes (Figure 6, B and D), with no significant differences observed. This suggests that the dosage of meiosis-related genes is sufficient for normal function in both genotypes at the time of birth, which potentially explains that the initial ovarian reserve was not in decline in *Mga^+/−^* females (Figure 5B). However, it is important to note that these meiosis-related genes displayed lingering expression in *Mga^+/−^* females, compared with the expected pattern of near-complete suppression shortly after birth observed in WT (Figure 6D). Taken together, the findings from ovarian bulk RNA-Seq suggest that the accelerated follicle depletion in *Mga^+/−^* mice might be attributed to the continual ectopic expression of meiosis genes resulting from MGA deficiency.

**Figure 7:**
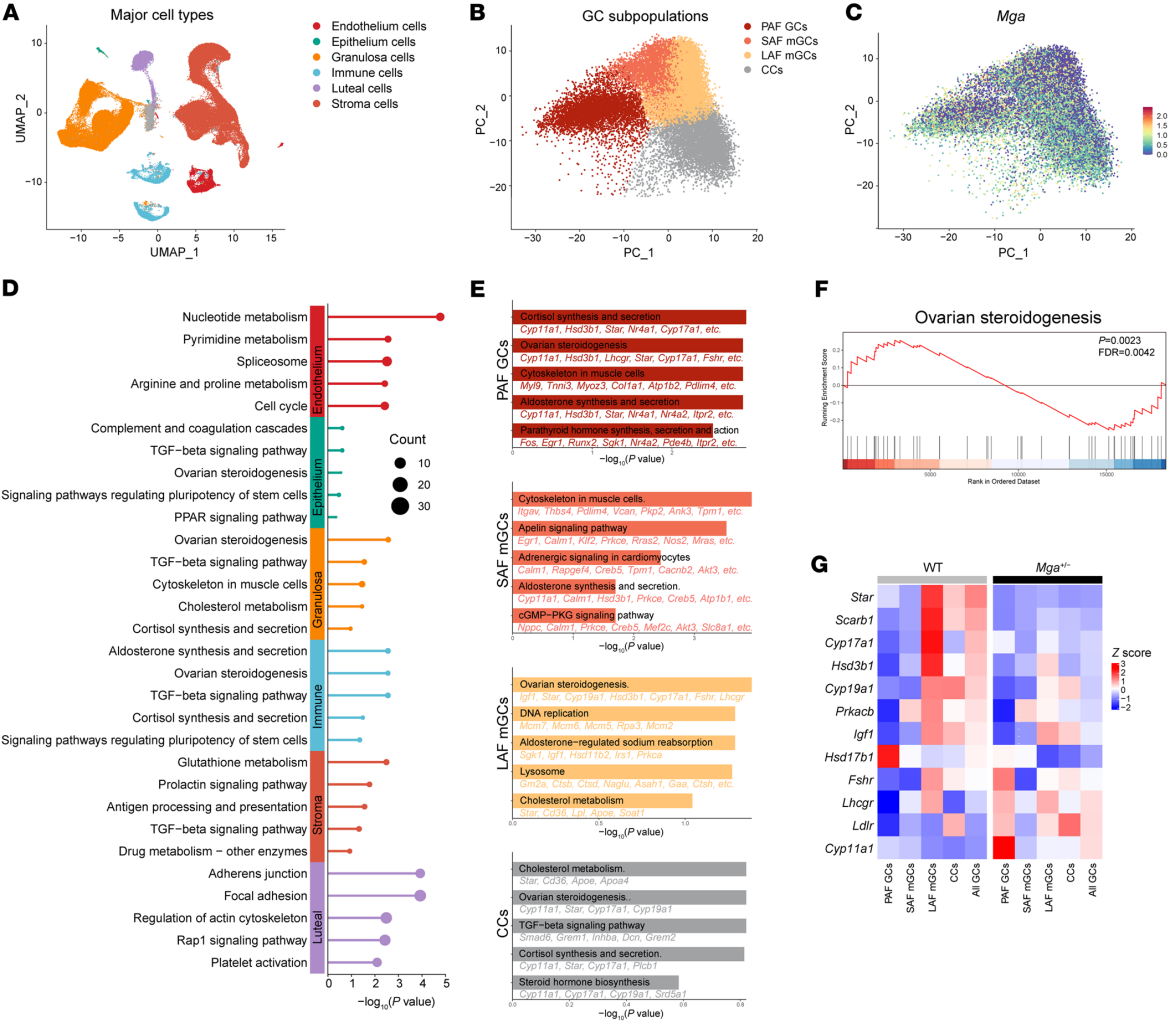
scRNA-Seq analysis uncovers altered functions in *Mga^+/−^* female mice. (**A**) Uniform manifold approximation and projection (UMAP) showing clusters of ovarian cell types for 6 major cell types. (**B**) PCA plot showing 4 subpopulations of GCs. (**C**) PCA plot showing the expression of *Mga* in GC subpopulations. (**D**) Pathway enrichment analysis using KEGG database of DEGs for 6 major cell types. (**E**) Bar chart showing KEGG pathway enrichment result in PAF GCs, SAF mGCs, LAF mGCs, and CCs. (**F**) GSEA showing enrichment of suppressed ovarian steroidogenesis in ovaries from *Mga^+/−^* female mice compared with WT female mice. (**G**) Heatmap showing the expression of DEGs involved in ovarian steroidogenesis in GCs.

To further characterize the effect of folliculogenesis in *Mga^+/−^* female mice, we conducted single-cell RNA-Seq (scRNA-Seq) on somatic cells from ovaries collected at 6 months of age, when fertility started to decline in *Mga^+/−^* female mice compared with their corresponding WT controls. We collected 95,169 cells and identified 19 clusters that we classified into 6 major cell types (Figure 7A and Supplemental Figure 7). GCs were further classified into 4 subpopulations: preantral follicle (PAF) GCs, small antral follicle (SAF) mural GCs (mGCs), the large antral follicle (LAF) mGCs, and the cumulus cells (CCs) (Figure 7B and Supplemental Figure 7D). Principle component analysis (PCA) showed *Mga* ubiquitously expresses in all subgroups of GCs (Figure 7C), suggesting its potential involvement in the development of ovary. Kyoto Encyclopedia of Genes and Genomes (KEGG) pathway enrichment analysis of DEGs showed that ovarian steroidogenesis was the most significant pathway in all GCs (Figure 7D) and was the top 3 pathway in PAF GCs, LAF mGCs, and CCs (Figure 7E). Gene set enrichment analysis (GSEA) revealed that the expression of genes involved in ovarian steroidogenesis is extremely disturbed (Figure 7F). Twelve DEGs involved in ovarian steroidogenesis were identified. The majority were downregulated across all subgroups of GCs, with the most pronounced difference observed in LAF mGC, while *Cyp11a1*, *Lhcgr*, and *Ldlr* were upregulated specifically within PAF mGCs (Figure 7G and Supplemental Figure 8). These findings are in line with the noted decrease in follicle numbers at various stages within the ovaries of *Mga^+/−^* females, suggesting that MGA deficiency could potentially influence ovarian development through the disruption of ovarian steroidogenesis, thereby introducing the POI-like phenotype.

## Discussion

Here, using standard gene-based collapsing analysis of WES data from a large sample size discovery cohort of POI patients with ethnically matched controls, we identified *MGA* as a candidate causal gene for POI, based on its highest significant enrichment. It was the only coding gene that showed exome-wide association between LoF variants and POI, accounting for 2.6% (27/1,027) of cases in the discovery cohort.

Furthermore, we also screened *MGA* LoF variants in 4 follow-up POI cohorts from China and the US. Ultimately, LoF variants in *MGA* were cumulatively responsible for 2.0% of total POI cases in this study, which is markedly higher than that of coding variants from any single known POI-associated gene (each <1.0%) and hundreds of times higher than carrier frequencies in the general population (around 1 in 10,000). These findings highlight the substantial role of *MGA* in the development of POI and its significance as a genetic factor in this condition.

The limited knowledge of *MGA* and the high genetic heterogeneity of POI may explain why *MGA* was not discovered in earlier POI studies. Primarily, the role of *MGA* in female fertility was largely unexplored prior to this study, with its absence of specific annotations related to ovarian development in widely utilized biological databases, such as GO, KEGG Pathway, and Reactome, leading to its exclusion from previous candidate gene studies on POI. Furthermore, although *MGA* represents the most significant genetic association signal, its contribution across POI cohorts is modest (1% to 2.6%), indicating that previous studies with limited sample sizes may have lacked the statistical power for robust findings; this also made it difficult to identify recurrent cases, especially with only around 100 patients. Our findings further emphasize the importance of large sample sizes and the advance of unbiased genome-wide or exome-wide association analyses in uncovering genetic factors underlying POI. This study also encounters the limitation of restricted sample sizes, particularly in POI cohorts from non–East Asian populations. To achieve a more accurate prevalence of *MGA* LoF variants in POI patients, future research will require larger cohorts from diverse ethnic groups.

Moreover, the potential involvement of other types of *MGA* coding variants in POI cannot be excluded. The frequencies of carrier with predicted damaging missense or in-frame variants did not significantly differ across the case cohorts and the control cohorts (Supplemental Table 6), suggesting that the alteration of 1 or a few amino acids in the protein may not be a major pathogenic mechanism of *MGA* in inducing POI. Some of these variants may be deleterious to the gene, but further functional assays are warranted to verify their impact. Additionally, the ability to detect other types of genetic variant, such as noncoding or structural variants, is limited by WES. Consequently, our current study may still underestimate the proportion of POI cases mediated by pathogenic *MGA* variants.

Previous studies have shown that *MGA* encodes a scaffold component of PRC1.6 that participates in suppressing germ cell–related gene expression in stem cells (34, 35). PRC1 complexes, including PRC1.6, regulate entry and exit from the meiotic prophase I program in female germline cells (36). Conditional knockout mice with dysfunctional PRC1 complexes in oocytes (PRcKO) exhibit total depletion of ovarian reserve and premature ovarian failure (36). Similarly, *Mga^+/−^* female mice displayed subfertility with accelerated depletion of ovarian reserve compared with WT mice. Ovaries of both female *Mga^+/−^* adult mice and PRcKO mice showed aberrant transcriptional upregulation of meiosis-related genes.

Prior studies on the etiology of POI have indicated that deficiencies in meiotic genes can serve as an important cause. However, the precise mechanisms by which the aberrant upregulation of meiosis-related genes could induce POI remain unclear. Our findings show that mouse orthologs of several known POI-causative genes, including *Meiosin*, *Rbm46*, *Stag3*, and *Syce1*, are significantly upregulated in ovarian tissues of *Mga^+/−^* adult mice, thus expanding our understanding of germ cell–related gene regulation in the pathogenesis of POI. The mechanisms of depletion of ovarian reserve caused by dominant POI genes are diverse, for example, inducing oocyte apoptosis by direct activating apoptosis factors (*TP63*) (37), excessive oocyte activation through ferroptosis (*BNC1*) (38), or loss of germ cells due to abnormal DNA damage repair (*FANCJ*) (39). It warrants mention that the reduced ovarian reserve in PRcKO mice has been previously proposed as an effect of cumulative toxicity caused by ectopic expression of meiosis genes or directly regulating apoptosis by regulating expression of apoptotic genes (36). However, we did not observe a significant upregulation of apoptotic genes in *Mga^+/−^* ovaries. The underlying mechanisms of potential toxicity or other possibilities resulting from MGA deficiency require future investigations using comprehensive functional experiments and oocyte-specific conditional knockout mice.

Heterozygous variants of dominant POI genes may not only independently affect POI, but also combine with additional deleterious variants in other POI-associated genes, acting as genetic modifiers (40). This combination may contribute to the observed differences in the severities of clinical manifestations among POI patients. Among the 27 cases with *MGA* LoF variants in the discovery cohort, 4 also harbored another heterozygous pathogenic/likely pathogenic (P/LP) variant in 4 other POI genes reported in our preliminary study, including *HFM1*, *RECQL4*, *SPIDR*, and *ERCC6* (Supplemental Table 7). Among them, *HFM1*, *RECQL4*, and *SPIDR* are known to cause POI through autosomal recessive inheritance. Therefore, individual heterozygous variants in *HFM1*, *RECQL4*, or *SPIDR* cannot alone serve as a direct genetic cause of POI, but potentially contribute to phenotypic variance among POI cases. The *ERCC6* variant (c.2839C>T, p.Arg947*) cooccurring with *MGA* LoF variant in the same patient affects the whole ERCC6 protein, but not the ERCC6-PGBD3 fusion protein. Intriguingly, in previously reported POI cases with heterozygous deleterious variants in *ERCC6*, whether affecting the entire ERCC6 protein or the ERCC6-PGBD3 fusion protein, all presented with secondary amenorrhea or spaniomenorhea (10, 41–43). However, proband POI-588 in our study, harboring heterozygous LoF variants in both *MGA* and *ERCC6*, presented with primary amenorrhea, further supporting the likelihood that genetic burden may affect severity of phenotypes.

The potential impact of *MGA* LoF variants on male infertility warrants further evaluation and investigation. In our POI cases, *MGA* LoF variants are paternally inherited in approximately half of the probands. Furthermore, no significant impairment of fertility was observed in young male mutant mice (Supplemental Figure 5), indicating that *MGA* LoF variants may not have a substantial impact on the fertility of young males. However, the proportions of *MGA* LoF variant carriers in men (44.2%) and women (55.8%) in gnomAD are not significantly different (*P* = 0.26, binomial test; Table 1), suggesting that these variants may also be subject to evolutionary selection in males. Most POI cases and female mice harboring *MGA* LoF variants exhibited a relatively mild phenotype, characterized by subfertility rather than completely infertility. Therefore, it is possible that males with *MGA* LoF variants also exhibit reduced fertility as they age compared with those without these variants. However, this hypothesis warrants further investigation through large-scale genetic screening in male subfertility/infertility cohorts and comprehensive fertility assessments of older male mice.

In summary, our findings from both human exome-wide studies in multicenter cohorts and functional studies in gene-edited mice strongly suggest that *MGA* LoF variants are a frequent genetic factor for POI and female subfertility through destabilization of the ovarian reserve.

## Methods

### Sex as a biological variable.

The association tests in humans were specifically based on female individuals. For human individuals from public databases, both sexes were included, but sex was not considered a biological variable due to the lack of sex information. Both male and female animals were used in this study, but the experiments were conducted and analyzed separately.

### Study participants.

Study patients diagnosed as having POI met the following criteria: (a) at least 4 months of oligo/amenorrhea occurring before age 40; (b) elevated serum follicle-stimulating hormone levels greater than 25 IU/L on 2 occasions (>4 weeks apart); and (c) no chromosomal abnormalities, iatrogenic cause (such as ovarian surgery/chemotherapy/radiotherapy), or history of autoimmune disorders. POI cases were further categorized into primary amenorrhea (absence of menarche before age 16 years) and secondary amenorrhea (spontaneous menstrual cycle at least once).

The study patients included multicenter cohorts from different countries and regions. The discovery cohort, referred to as cohort 1, initially comprised 1,050 women with POI diagnosis recruited from the Reproductive Hospital Affiliated to Shandong University (11). Four additional POI cohorts were enrolled for replication studies. The Southern Chinese cohort (i.e., cohort 2) was recruited at Nanfang Hospital, Southern Medical University, and comprised 196 unrelated POI cases. The Central Chinese cohort (i.e., cohort 3) was recruited at Tongji Hospital, Huazhong University of Science and Technology, and comprised 485 POI cases. The fourth cohort (i.e., cohort 4), which comprised 104 POI cases, was approved by Pittsburgh University, Pittsburgh, Pennsylvania, USA (44). The fifth cohort (i.e., cohort 5) was recruited at the Eunice Kennedy Shriver National Institute of Child Health and Human Development, NIH, Bethesda, Maryland, USA and included 98 cases with nonsyndromic POI (dbGaP accession number phs001174.v1.p1) (45).

### Exome sequencing.

WES was conducted in members of cohort 1 and cohort 2 using the AIExome V1-CNV capture kit (iGeneTech), with average coverages of ×114 and ×108, respectively. For cohort 3, exomes were captured using the Agilent SureSelect Human All Exon V6 Capture Kit with an average coverage of ×30. For cohort 4, exomes were captured using the Agilent SureSelect Human All Exon V4 or V5 Kit or NimbleGen SeqCap EZ Human Exome V3.0 with coverages from ×150 to ×250. For cohort 5, exome was captured using the NimbleGen VCRome 2.1 (HGSC design). Cohorts 1–3 were sequenced on the NovaSeq platform (Illumina), and cohorts 4 and 5 were sequenced using the Illumina HiSeq 2500. The above summary statistics are listed in Supplemental Table 1.

### Variants calling and annotation.

Raw sequencing data underwent cleaning using Fastp with default parameters (46). Subsequently, cleaned reads were aligned to the human reference genome GRCh37/hg19 using the Burrows-Wheeler Aligner (BWA 0.7.17) with the MEM algorithm (47). Following alignment, reads underwent preprocessing, and genotypes were jointly called for all samples within each cohort, following the best practices outlined in the Genome Analysis Toolkit (GATK 4.1.8.1) (48). Finally, variants were annotated using the Ensembl Variant Effect Predictor (VEP 102) with the corresponding RefSeq database (49).

### QC of samples.

Sample pruning was conducted in cohort 1 and its corresponding control cohort (2,739 women from the HuaBiao Project) (11, 19). Samples with an average depth of less than ×30 within coding regions were excluded. Relatedness of cases were accessed by KING (50). In pairs were assigned to Dup/MZTwin or 2nd degree; the one with low coverage was excluded. Twenty individuals were removed at this step (Supplemental Figure 1).

To address population stratification and ascertain the genetic ancestry of individuals, principal component analysis was conducted across cases and controls in the discovery cohort, using individuals from the 1000 Genomes Project Phase III as a reference for ancestry indicators (Supplemental Figure 2A). Samples falling within ±4 standard deviations across principal components 1 and 2 were retained for subsequent association tests, which resulted in 9 ethnicity outliers (3 cases and 6 controls) of East Asian ancestry using principal component analysis (Supplemental Figure 2B). Finally, 1,027 POI cases from cohort 1 and 2,733 matched women controls from the HuaBiao Project served as controls (19) and were used in the subsequent association analysis.

### QC of variants.

To minimize bias in association tests, qualifying variants (QVs) were selected for association tests according to their sequencing quality, mapping quality and read depth. Variants met the following criteria: (a) mean read depth (DP) >10; (b) alternative allele read frequency ≥25%; (c) mean quality by depth (QD) >10; (d) mean phred quality (QUAL) >30; (e) mean genotype phred quality (GQ) >20. Coverage harmonization analysis of exons was not performed, as both cases and controls in the discovery cohort were subjected to the same exome capture kit and sequencing platform.

### Gene burden tests.

To evaluate whether specific genes were significantly enriched in cases of POI when compared with controls across the entire exome, a standard gene-based collapsing analysis was executed (15). The analysis focused on rare variants with an MAF of less than 0.1% within the internal case and control cohorts, as well as external population cohorts. This threshold was based on the expectation that variants significantly impacting reproductive capability would not be common in the general population. LoF variants encompassed variants leading to protein truncation, including canonical splice-site variants, frameshift variants, and nonsense variants. Damaging missense variants were defined as predicted to be deleterious by SIFT2, Polyphen2, and MutationTaster. Synonymous variants, which are typically considered functionally neutral, were used to assess whether there is a preferential inflation of background variation.

A final set of 19,199 protein-coding genes, which have at least one rare LoF, damaging missense, or synonymous variant, were subjected to testing using canonical transcripts for each gene. For each gene, associations were calculated using 2-sided Fisher’s exact tests, chosen for its robustness and absence of test statistic inflation in sparse data (51). To obtain the expected distribution of *P* values, 1,000 permutations for relabeling case and control were performed. The Bonferroni’s adjusted significance threshold for each model was set at 0.05/19,199 = 2.6 × 10^−6^. The λ factor for the quantile-quantile (Q-Q) plot was calculated using the slope of the observed versus expected values obtained through linear regression, excluding *P* values of 1.0 or those exceeding the Bonferroni’s correction threshold.

### Minigene splicing assay.

Minigene splicing assays were conducted to validate the impact of splice-site variants in *MGA*, including c.5504-2A>G, c.7139+1G>A, and c.7008+1G>A. WT fragments flanking the variant locations with restriction sites (*Kpn*I and *Eco*RI) were obtained from human genomic DNA through nested PCR amplification. The WT fragments were as follows: c.5504-2A>G: exon 16 (291 bp); intron 16 (183 bp); exon 17 (1505 bp); 5′ terminal intron 17 (199 bp); c.7139+1G>A: exon 18 (131 bp); 5′ terminal intron 18 (644 bp); c.7008+1G>A: exon 16 (219 bp); intron 16 (183 bp); exon 17 (1505 bp); and 5′ terminal intron 17 (379 bp).

The corresponding variants were introduced through overlap extension PCR to generate mutant fragments. Subsequently, WT or mutant fragments were digested and ligated into the pcMINI-N vector (Bioeagle Biotech Company), facilitating the creation of both WT and mutant constructs. These constructed plasmids were transfected into HeLa and 293T cell lines. After 48 hours, cells were harvested, and total RNAs were extracted using TRIzol reagent (Invitrogen). The extracted RNA was reverse transcribed into cDNA using the genome DNA PrimeScript RT Reagent Kit (Takara Biomedical Technology). The cDNA was then amplified by PCR using primers designed to flank the minigene region of interest. The resulting PCR products underwent separation via agarose gel electrophoresis. Each band was gel purified, and these purified fragments were subjected to sequencing to determine the transcripts produced by the WT and mutant constructs. The primers used in minigene assays are provided in Supplemental Table 8.

### Generation of Mga^+/−^ mice.

We generated the *Mga*-mutated mouse model using the CRISPR/Cas9 gene-editing technique by directly injecting CRISPR/Cas9 reagents into zygotes. Cas9 was amplified from the pX260 plasmid (Addgene) using KOD-Plus-Neo (KOD-401, TOYOBO) and purified using the Universal DNA Purification Kit (DP214, TIANGEN). The purified Cas9 was then transcribed in vitro using the mMESSAGE mMACHINE T7 ULTRA Kit (AM1345, Life Technologies). The single-guide RNA (sgRNA) containing a T7 promoter was amplified from the pX330 plasmid (Addgene) using KOD-Plus-Neo polymerase and purified using the Universal DNA Purification Kit. The purified sgRNA was then transcribed in vitro using the MEGAshortscript T7 Kit (AM1354, Life Technologies). Following transcription, both Cas9 and sgRNA were purified using the MEGAclear kit (AM1908, Life Technologies) according to the manufacturer’s instructions.

Zygotes were collected as previously described (52). The Cas9/sgRNA complex was injected into the zygotes, and the embryos were cultured in EmbryoMax KSOM Mouse Embryo Media (Merck Millipore) at 37°C with 5% CO_2_ until the 2-cell stage. Subsequently, the embryos were transferred into the oviducts of pseudopregnant female mice at 0.5 days postcoitum. Founder mice harboring a frameshift variant in *Mga* were selected and mated with C57BL/6 mice to establish a stable mouse line with the *Mga* mutation.

We used 2 approaches to check for off-target effects of CRISPR. First, according to CRISOT (53), the sgRNA used to edit *Mga* has a CRISOT score of zero, indicating a safe target sequence. Second, CRISPRater was used to predict off-target sequences (54). Sanger sequencing of these predicted regions detected no variants at these loci in the gene-edited mice.

### Fertility assessment of mice.

To assess the fertility of female mice, each adult male with WT genotypes aged 8–10 weeks was mated with 1 *Mga^+/−^* female and 1 WT female aged 8 weeks. This mating scheme was implemented over the course of 1 year. A total of 8 groups were formed. Throughout the mating period, the ages of the females and the number of pups in each litter were meticulously recorded.

To assess the fertility of male mice, spermatozoa were collected from the epididymides of adult male mice at 8−10 weeks and were diluted in 1 mL human tubal fluid (HTF, MR-070-D, Millipore) for 15 minutes at 37°C. Sperm concentration and mobility were evaluated using a computer-assisted sperm analysis system with spermatozoa obtained from the cauda epididymides.

### 3D reconstruction of ovaries in mice.

In this study, we employed the Clear, Unobstructed Brain/Body Imaging Cocktails and Computational analysis (CUBIC), a well-established tissue-clearing and imaging technique, which has previously found success in mouse brain, ovary, and whole-body studies (55–58). Our aim was to quantify the whole ovarian reserve at the single-oocyte level by reconstructing 3D ovaries using the CUBIC method. This process encompassed 3 primary steps: tissue clearing, immunofluorescence staining, and imaging scanning for oocyte counting.

### Tissue clearing.

Initially, deeply anesthetized mice underwent perfusion with 10 ml of cold PBS to remove blood from the ovaries. Subsequently, following decapitation, the ovaries were dissected and fixed in 4% paraformaldehyde for a 24-hour period. After fixation, the ovaries underwent dehydration using a 30% sucrose solution for another 24 hours. Following dehydration, the ovaries were washed with PBS in preparation for the subsequent CUBIC clearing process. We prepared 2 CUBIC reagents (CUBIC-I and CUBIC-II) following the established protocol (29). The ovaries were cleared in CUBIC-I reagent at 37°C for 7–10 days until they achieved transparency. The ovaries were then washed in PBS until all bubbles dissipated.

### Immunofluorescence staining.

The cleared ovaries were blocked in PBS containing 10% Triton X-100 and 10% donkey serum, undergoing agitation at 37°C for 3 days. Following this, the ovaries of P0 were incubated with primary antibodies against TRA98 (1:1000, Abcam, ab82527), and the ovaries at age 4M and 8M were incubated with primary antibodies against DDX4 (1:1000, Abcam, no. ab13840) and P63 (1:5000, Abcam, no. ab53039), which label the plasma and nuclei of oocytes, respectively. This incubation took place on a 37°C shaker for an additional 3 days. After 5 washes with PBS (1 hour per wash), the ovaries were incubated with the secondary antibody (1:1000, Invitrogen, no. A10520, goat anti-rabbit IgG (H+L) cross-adsorbed secondary antibody, cyanine3) and DAPI for an additional 3 days. Following another 5 washes with PBS, the stained ovaries were treated with CUBIC-II reagent for 24 hours at 37°C to adjust refractive indices.

### Imaging scanning and oocyte counting.

Each stained ovary was positioned on the microscope stage, immersed in CUBIC-II reagent, and surrounded by butyl rubber. The Nikon A1 confocal system captured fluorescence 3D images of the ovaries, with a Z-series step size of 3 μm. Oocyte counting was then conducted automatically using the Spots algorithm of the Imaris ×64 9.5.0 Image Workstation, followed by manual verification. Oocytes with fluorescent spot diameters exceeding 40 μm were identified as large oocytes, indicative of growing oocytes, while oocytes with spot diameters between 14 and 40 μm were classified as small oocytes, representing quiescent oocytes.

### H&E staining and follicle counting of ovaries in mice.

Ovaries were fixed in 4% paraformaldehyde for over 24 hours and then were dehydrated and embedded in paraffin. Subsequently, the embedded ovaries were sectioned at a thickness of 5 μm and every fifth section was stained with H&E. The histological analysis was conducted as previously described. Briefly, follicles containing an oocyte with a clearly visible nucleus were counted, and the classification of follicles into different developmental stages was performed according to the system established by Pedersen and Peters (59).

### Bulk RNA-Seq and analyses of mouse ovaries.

Total RNA was extracted from the ovaries of P1, P5, 1M, and 5M female mice and isolated using the RNeasy Mini Kit (QIAGEN). A fraction of the extracted total RNA (400 ng) underwent processing into strand-specific libraries using the TruSeq Stranded Total RNA Sample Preparation Kit (Illumina), following the manufacturer’s protocol. The purified libraries were quantified using a Qubit 2.0 Fluorometer (Life Technologies). To ensure library quality and integrity, the insertion size and mole concentration were validated using an Agilent 2100 bioanalyzer (Agilent Technologies). The purified libraries were sequenced on NovaSeq 6000 (Illumina). Raw sequencing data of reads were cleaned using Fastp, followed by alignment to the mouse reference genome GRCm38/mm10 using STAR with default parameters (46, 60). Read counts of genes were calculated using featureCounts (version 2.0.1) (61). Genes with |log_2_FC| > 1 and FDR (i.e., *P* value adjusted by the Benjamini-Hochberg method) < 0.05 were considered DEGs. Normalization and statistical analyses for DEGs used DESeq2 (62). GO enrichment analyses of DEGs were conducted by an online functional annotation clustering tool, Metascape (63).

### scRNA-Seq and analyses of mouse ovaries.

The ovaries of female mice age 6M were isolated and were dissociated using trypsin (Gibco, Thermo Fisher Scientific, no. 15090046), collagenase II (Gibco, Thermo Fisher Scientific, no. 17101015), and DNase I (Applichem, no. A3778.0050). Digested cells were subsequently filtered using a 40 μm strainer (Falcon, no. 352340). After treatment by red blood cell lysis solution (MACS, no. 130-094-183), cells were washed and suspended using DMEM medium (Gibco, Thermo Fisher Scientific, no. 11995065). Cell counting, concentration and viability were assessed by LUNA-II Automated Cell Counter, and the fresh cell suspension was adjusted to a cell concentration of 700–1200 cells/μl. Then, the scRNA-Seq libraries were prepared using 10× Genomics Chromium Next GEM Single Cell 3′ Reagent Kits, version 3.1 (no. 1000268), according to the manufacturer’s instructions and were sequenced on an Illumina NovaSeq 6000 with PE150 read length.

The raw sequencing data were cleaned and aligned to the mouse reference genome GRCm38/mm10 using Cell Ranger, version 7.0.1 (10X Genomics) with default parameters to generate the unique molecular identifier (UMI) count matrix summarized for each barcode. The further analyses, including filtering, data normalization, dimensionality reduction, and clustering, were conducted using Seurat, version 5.0.3 (64). For the clustering and subtype analysis of the total cell population, the Louvain algorithm was implemented at a resolution of 0.3, identifying 19 distinct clusters (Supplemental Figure 7A). Then the clusters were annotated using cell-type–specific signatures and marker genes into 6 major cell types: endothelium cells, epithelial cells, GCs, immune cells, luteal cells, and stroma cells (Supplemental Figure 7B) (65). No cluster could be classified to oocytes (Supplemental Figure 7C). For further classifying subpopulations of GCs, PCA was performed; the K-means algorithm from the stats package (version 4.3.2) was utilized to define clusters. According to the expression of marker genes (66), GCs were further classified into 4 groups: PAF GCs, SAF mGCs by *Cald1*, the LAF mGCs by *Inhba*, and the CCs by *Top2a* (Supplemental Figure 7D).

DEGs were defined as those genes with |log_2_FC| > 0.5 and FDR < 0.05. KEGG enrichment analysis was performed on DEGs in each cell type of GCs and all GCs using hypergeometric distribution. GSEA was conducted by clusterProfiler (67) to compare the expression difference of genes involved in the ovarian steroidogenesis signaling pathway between 2 genotypes.

### Statistics.

Data were analyzed using R 3.6.0. The experimental results between 2 groups were compared by 2-tailed Student’s *t* test. A *P* value of less than 0.05 was considered significant.

### Study approval.

Informed consent was obtained from each participant, and the appropriate institutional forms have been archived. This study was approved by the collaborating hospitals and the Ethics Committee of Obstetrics and Gynecology Hospital, Fudan University. All animal experiments were conducted with the prior approval of the Animal Care and Use Committee at Fudan University, strictly adhering to its ethical guidelines.

### Data availability.

The sequencing data of cases in cohort 1 in this study have been deposited in the Genome Sequence Archive (GSA) in the National Genomics Data Center, China National Center for Bioinformation/Beijing Institute of Genomics, Chinese Academy of Sciences. The accession number of these data is HRA003245, publicly accessible at https://ngdc.cncb.ac.cn/gsa-human/ The data for cohort 5 were accessed through the NCBI’s dbGaP (phs001174.v1.p1). The data for cohorts 2, 3, and 4 in the current study are available upon request. The control data used in the discovery stage of our study is derived from the HuaBiao project, which is deposited in the National Omics Data Encyclopedia under the accession number OEZ00008311 (https://www.biosino.org/node/analysis/detail/OEZ00008311). As for the data of the controls used in the follow-up stage, we retrieved and downloaded genomic variants of MGA (hg38: chr15:41,660,397-41,773,081; hg19: chr15:41,952,610-42,062,141) from the websites of public databases, and re-annotated them using VEP 102. The public databases used in analyses can be obtained from the following links: BRAVO: https://bravo.sph.umich.edu/; ChinaMAP: www.mbiobank.com; gnomAD: https://gnomad.broadinstitute.org/; HuaBiao project: https://www.biosino.org/wepd/; NyuWa Genome resource: http://bigdata.ibp.ac.cn/NyuWa_variants/; Regeneron Genetics Center: https://rgc-mcps.regeneron.com/ The data values for Figure 4, Figure 5, and Supplemental Figure 5 are provided in the Supporting Data Values file.

## Author contributions

ST, FZ, and ZJC designed the study. ST analyzed the data of WES, bulk RNA-Seq, and scRNA-Seq and the results of experiments. ST and FZ wrote the manuscript. YQ, TG, SW, Y Li, JZ, SC, and AR provided ascertainment, recruitment, and phenotypic characterization of patients. WT accessed and analyzed the data from dbGaP. HK, SZ, Y Li, WW, Y Lai, and AW performed Sanger sequencing validation. LW and BW performed the generation and maintenance of gene-edited mice. CS and XL performed the fertility assessment and the quantitation of oocytes in mice. Y Liu processed and analyzed the data of scRNA-Seq. SX and LJ contributed to variant calling and quality control of the control data. All authors reviewed the manuscript and are in agreement for manuscript publication. The order of co–first authors was determined on the basis of ST’s contributions to the work (leading the majority of the analyses performed and writing of the manuscript).

## Supplementary Material

Supplemental data

Supporting data values

## Figures and Tables

**Table 1:**
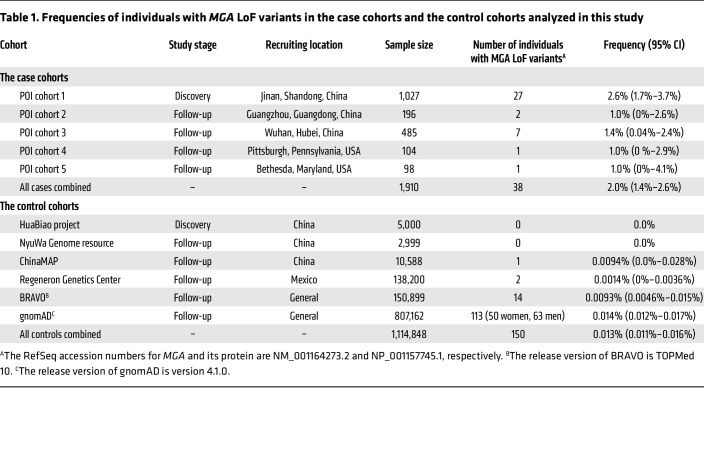
Frequencies of individuals with *MGA* LoF variants in the case cohorts and the control cohorts analyzed in this study

## Version 1 11/15/2024

Electronic publication
