# Supplementary material for: MGA loss-of-function variants cause premature ovarian insufficiency
Source: J Clin Invest. 2024 Nov 15;134(22):e183758. doi: 10.1172/JCI183758 (PMC11563689; doi:10.1172/JCI183758)
Supplement: Supplemental data [file jci-134-183758-s054.pdf]

Supplemental Figures

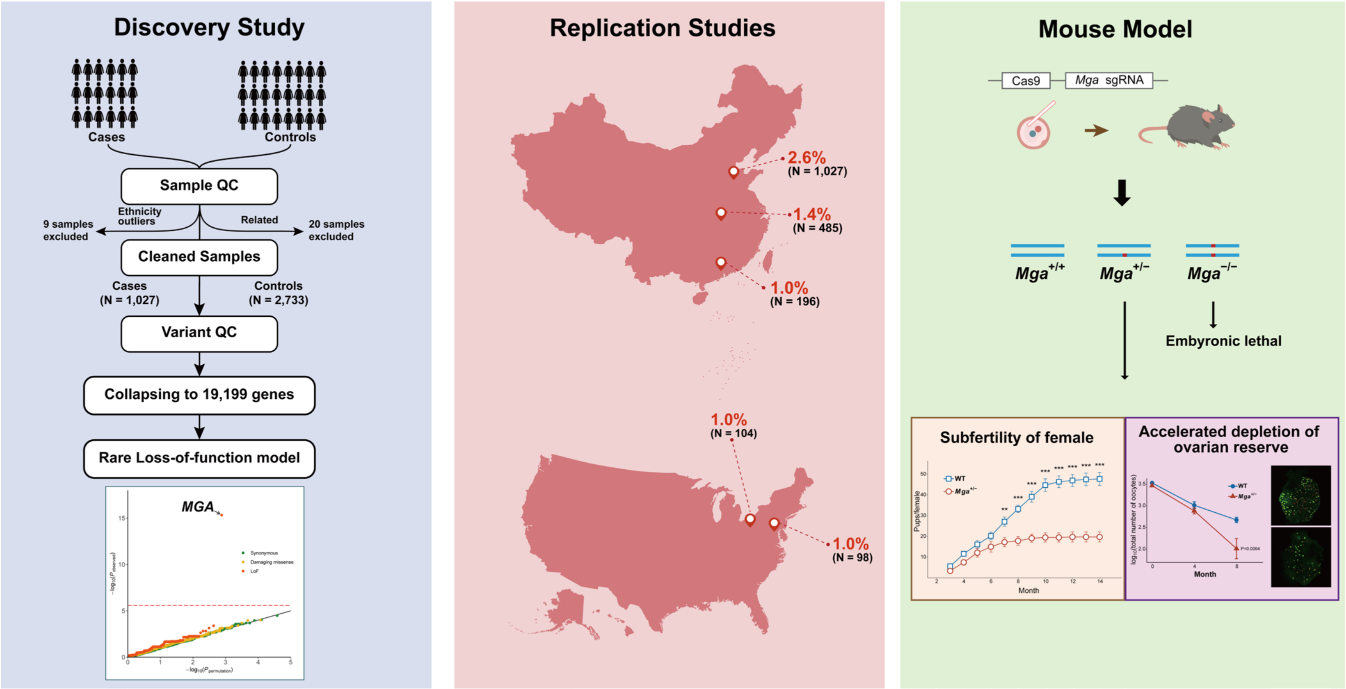

Supplemental Figure 1. Study design. The study commenced with a discovery phase, during which we conducted a standard gene-based collapsing analysis using a large POI cohort from Northern China and ethnically matched controls. In this analysis, we identified the gene with the highest enrichment of loss-of-function (LoF) variants, which turned out to be *MGA*. To validate our initial finding, we screened for *MGA* LoF variants in four additional follow-up POI case cohorts from different regions: Central China (N = 485), Southern China (N = 196), and Pittsburgh (N = 104) and Bethesda (N = 98) in United State of America. In these cohorts, we observed respective *MGA* contributions of 1.4%, 1.0%, 1.0%, and 1.0% among the cases. To establish a causal relationship between heterozygous *MGA* LoF variants and POI, we generated a gene-edited mouse model by introducing a heterozygous frameshift mutation into *Mga* ($Mga^{+/-}$) to mimic *MGA* LoF variants in humans. $Mga^{+/-}$ female mice displayed reduced fertility and an accelerated depletion of ovarian reserve, effectively replicating the POI phenotype observed in women. Abbreviations: QC, quality control.

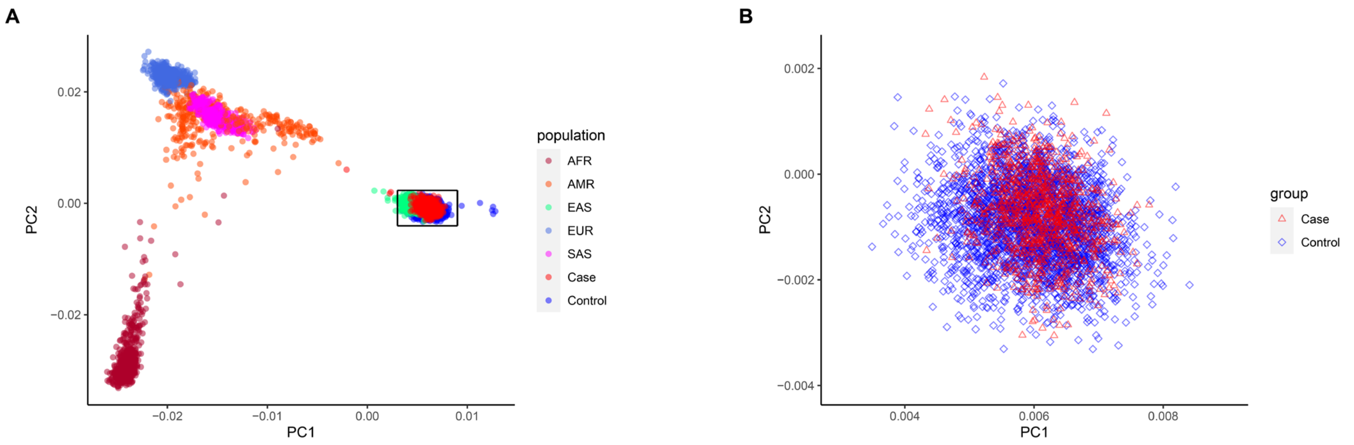

Supplemental Figure 2. Principal component analysis for human subject ancestries in the discovery phase. (A) Scatter plot illustrating the distribution of individuals from the discovery cohort, encompassing 1,030 unrelated cases (in red) and 2,739 controls (in blue), concerning principal components (PC) 1 versus 2. The plot also incorporates individuals representing various ancestries from the 1000 Genomes Project Phase III. To ensure the homogeneity of our cases and controls, individuals were retained only if they did not exceed 4 standard deviations along any principal component. **(B)** Ultimately, 1,027 cases and 2,733 controls were retained in the analysis. Abbreviations: AFR, African; AMR, Ad Mixed American; EAS, East Asian; EUR, European; SAS, South Asian.

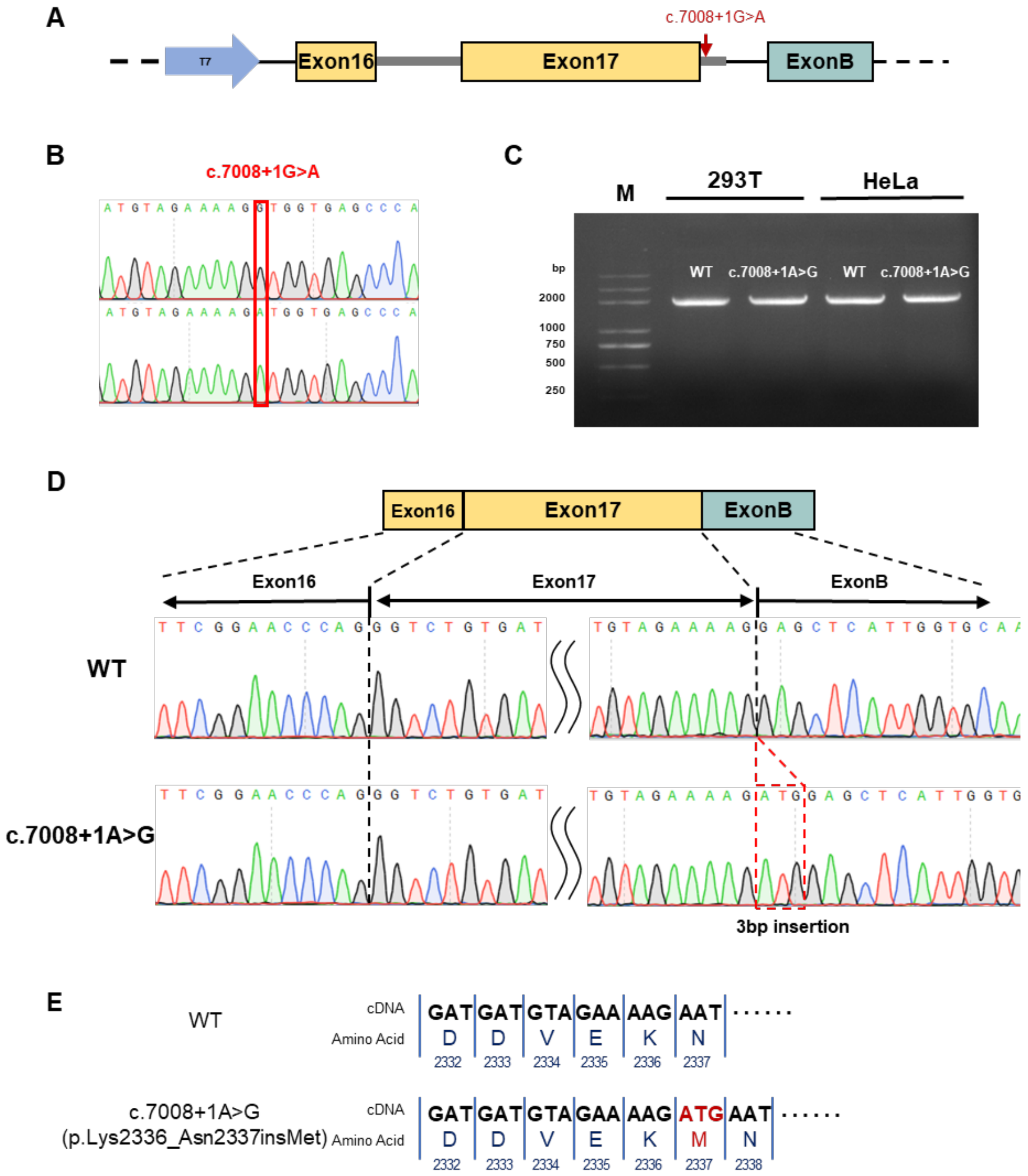

Supplemental Figure 3. Splicing results of the *MGA* c.7008+1G>A variant from general population by mini-gene assays. (A) Schematic representation of the mini-gene assay strategy used to investigate splicing effects of the *MGA* c.7008+1G>A variant. **(B)** Sanger sequencing results of the recombinant vector. Upper: wild-type (WT); lower: mutant. **(C)** Electrophoresis results showing the transcript PCR products obtained from both 293T and HeLa cell lines. The PCR products show similar length between WT and the mutant. **(D)** Splicing results comparing *Mga* WT and c.7008+1G>A variant. Sanger

sequencing of the PCR products reveal that the transcript of the c.7008+1G>A variant lost the original donor site and gained a new donor site at c.7008+4 in intron 17. **(E)** The introduction of the new donor site at c.7008+4 in intron 17 results in the incorporation of an additional amino acid in the protein. Consequently, the c.7008+1G>A variant causes an in-frame variant instead of protein truncation.

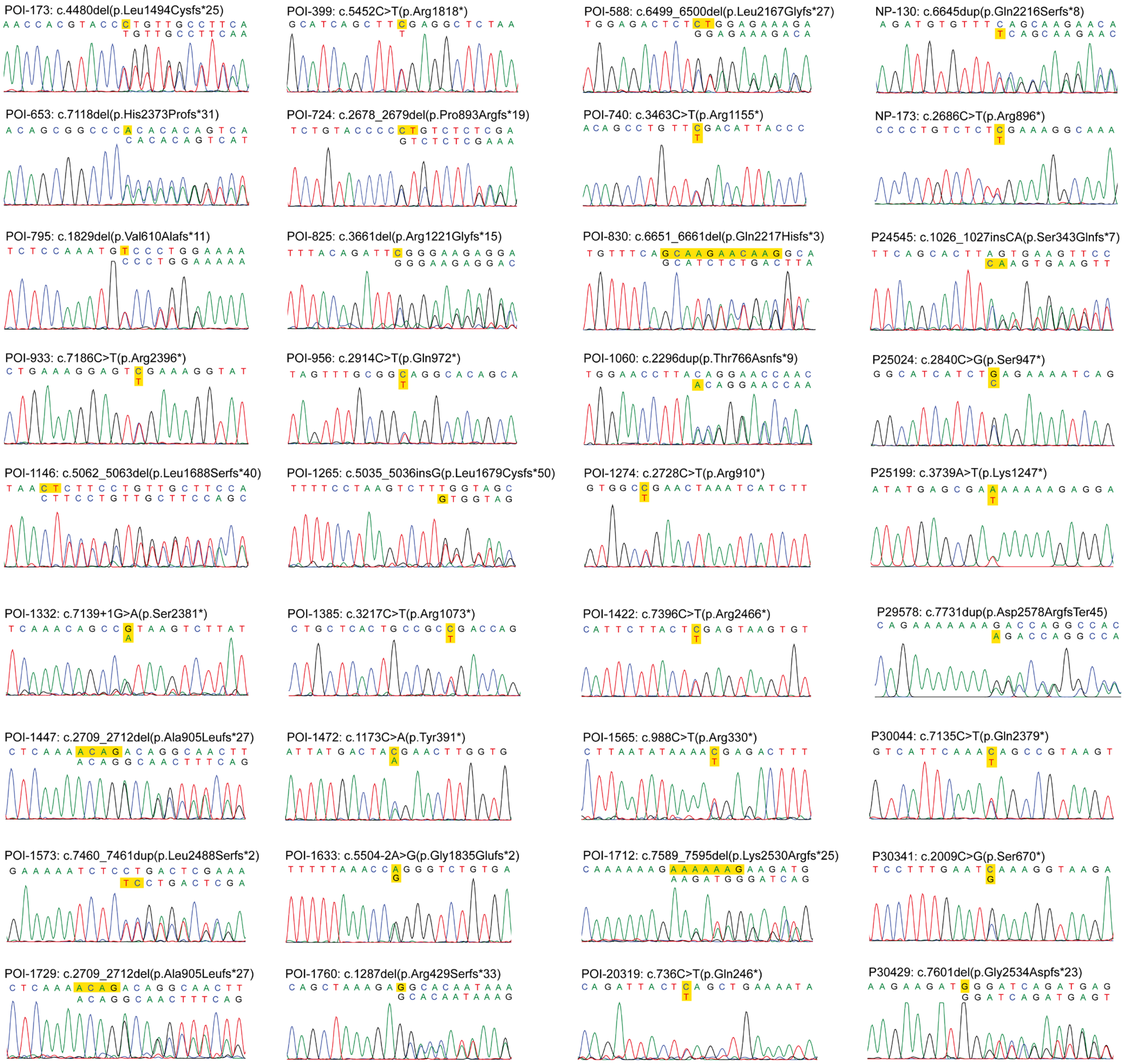

Supplemental Figure 4. Sanger sequencing verified the LoF variants of *MGA* in POI patients. The patients were confirmed to harbor a heterozygous allele of the identified LoF variant. The mutated nucleotides are highlighted.

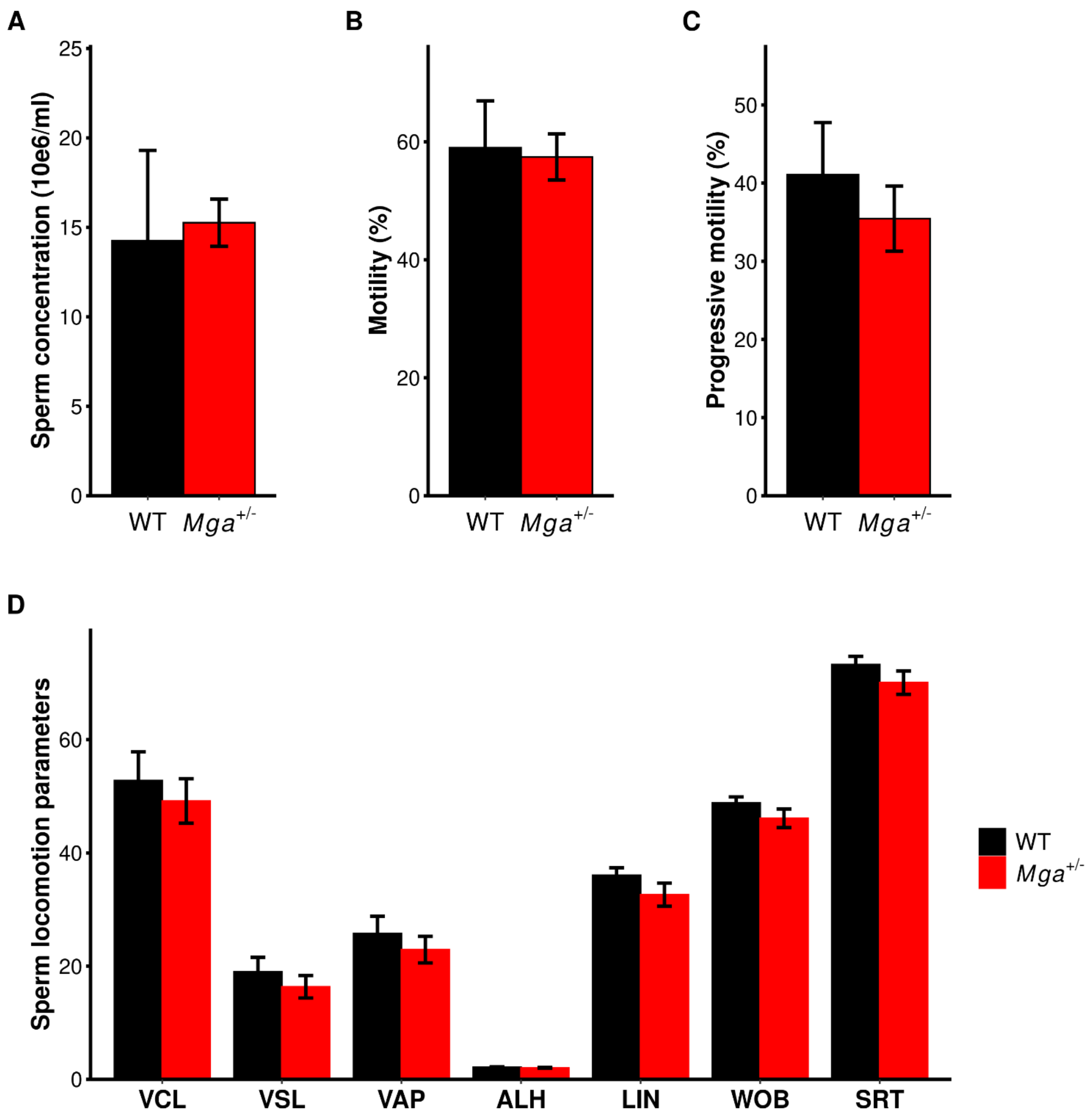

Supplemental Figure 5. Semen characteristics of male mice via computer-assisted analysis system. **(A)** Concentration, **(B)** motility, and **(C)** progressive motility of sperm from *Mga*$^{+/-}$ male mice (n=6) were compared to those of wild-type (WT) male mice (n=4). No statistically significant difference was observed. **(D)** Sperm locomotion parameters, including curvilinear velocity (VCL; µm/s), straight-line velocity (VSL; µm/s), average-path velocity (VAP; µm/s), amplitude of lateral head displacement (ALH; µm), linearity of the curvilinear path (LIN; %), sperm wobbliness (WOB=VAP/VCL; %), straightness of the average path (SRT; %), and average motion degree (MAD; °), were compared between WT and *Mga*$^{+/-}$ male mice. No statistically significant differences in these parameters were observed. Error bars represent the standard error of means.

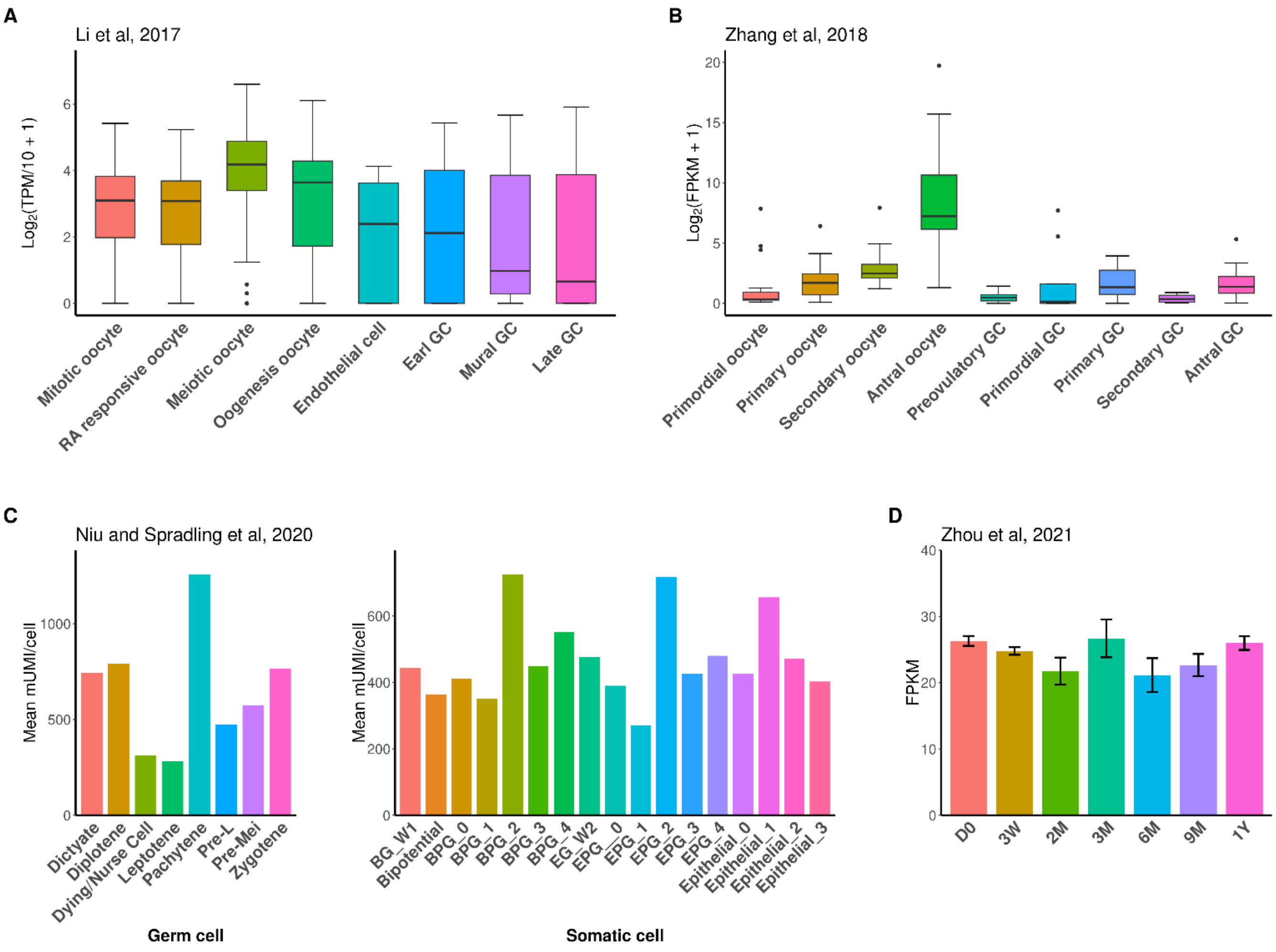

Supplemental Figure 6. ***MGA*** **mRNA expression in female germ cells and somatic cells of human and mouse. (A)** *MGA* mRNA expression in female germ cells and somatic cells of human fetal ovaries. The single-cell RNA sequencing (scRNAseq) data were derived from Li *et al.* (1). **(B)** *MGA* mRNA expression in female germ cells and granulosa cells of human adult ovaries. The scRNAseq data were derived from Zhang *et al*. (2) Boxplots depict the minimum, the 25th percentile, the median, the 75th percentile, and the maximum. **(C)** *Mga* mRNA expression in female germ cells and somatic cells of mouse embryo ovaries. The scRNAseq data were derived from Niu and Sprading *et al.* (3). **(D)** *Mga* mRNA expression in mouse ovaries at different development stages, including postnatal day 0 (D0), 3 weeks (3W), 2 months (M), 3M, 6M, 9M, and one year (1Y). The data was sourced from Zhou *et al.* and generated by bulk RNAseq(4) Abbreviations: BG_W1, bipotential granulosa wave 1; BPG, bipotential preganulosa cells; EG_W2, epithelial granulosa wave 2; EPG, epithelial preganulosa cells; GC, granulosa cell.

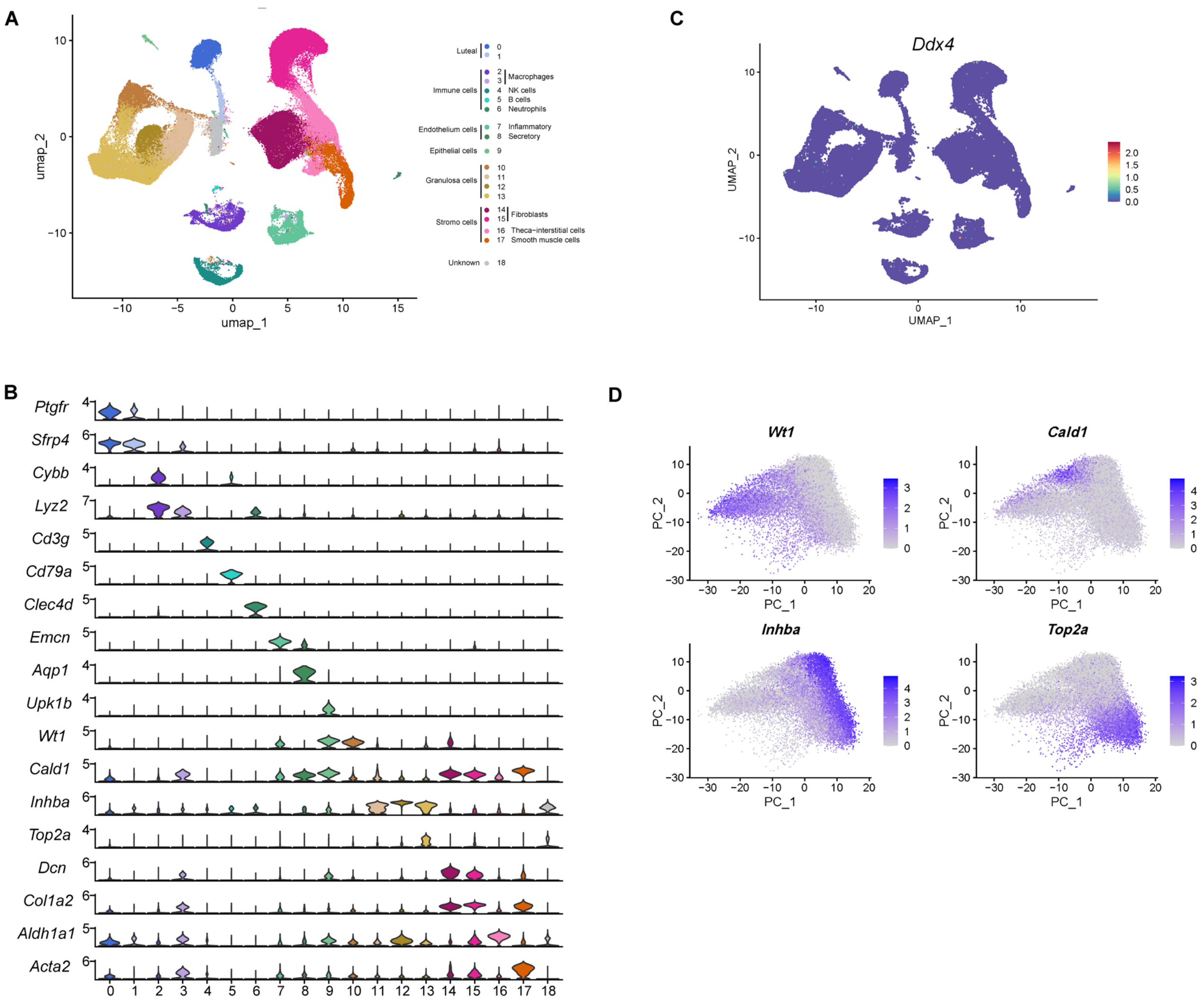

Supplemental Figure 7. Clusters of single-cell RNAseq in somatic cells of mouse ovaries. (A) UMAP plot showing 19 clusters and their corresponding cell types. **(B)** Violin plots showing expression of one representative differential expressed gene for each cluster. **(C)** UMAP plot showing the expression of *Ddx4*. None of cluster can be defined as oocytes. **(D)** PCA plots showing expression of selected granulosa cell marker genes.

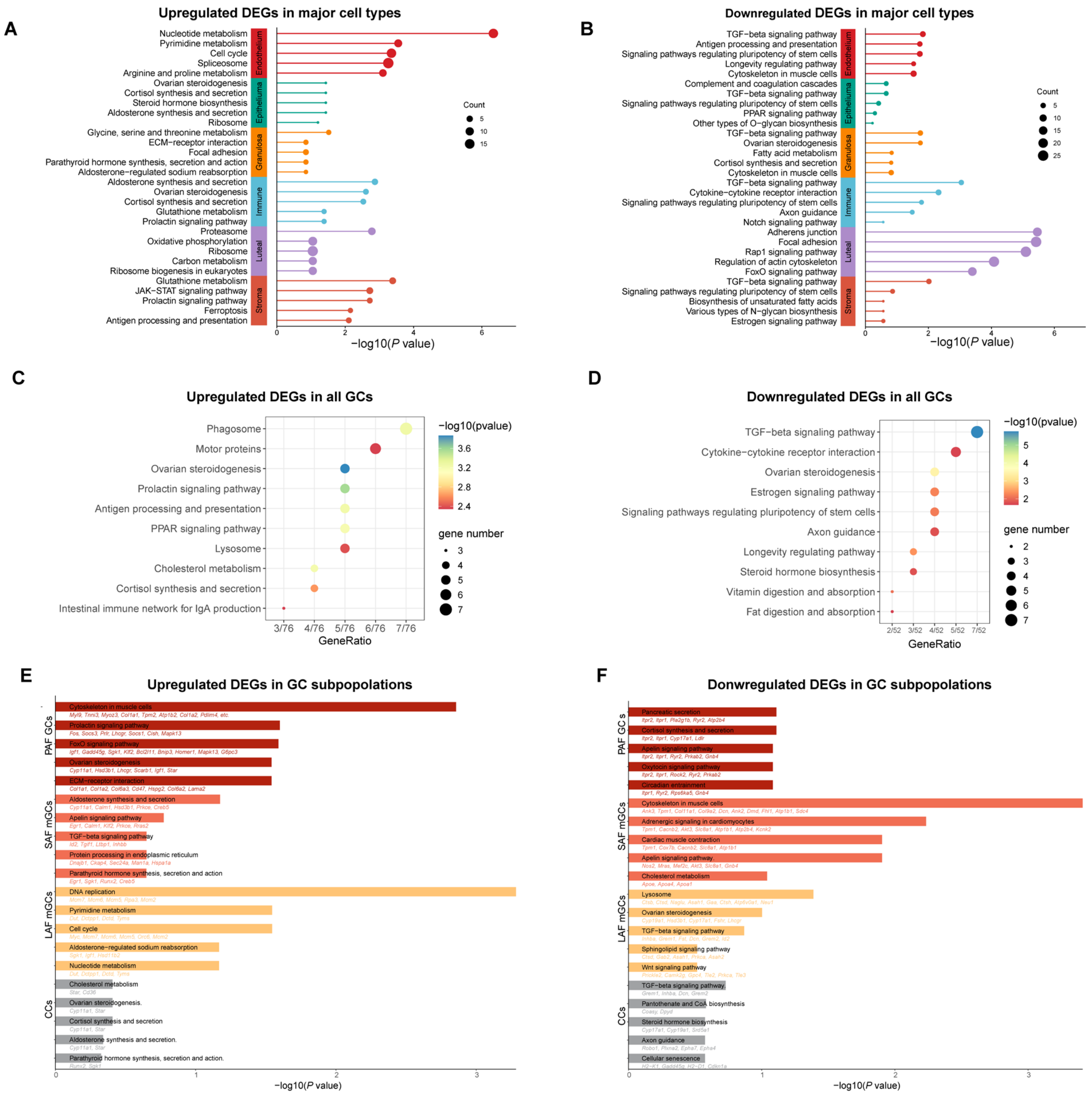

Supplemental Figure 8. Enrichment results of differential expression genes from single-cell RNAseq. (A, C, E) KEGG pathway enrichment results of upregulated differential expression genes (DEGs) in all cells **(A)**, six major cell types **(C)**, and four subpopulations of granulosa cells **(E)**. **(B, D, F)** KEGG pathway enrichment results of downregulated DEGs in all cells **(B)**, six major cell types **(D)**, and four subpopulations of granulosa cells **(F)**.

Supplemental Tables

Supplemental Table 1. Sequencing summary of case cohorts and control cohorts.

Group	Cohort	Study stage	Capture methods	Sequencing platform	Mean coverage of whole exome/genome (×)	Mean coverage of *MGA* (×)
Case cohorts	Cohort 1	Discovery	iGeneTech ALExome V1-CNV	Illumina NovaSeq	114	105
	Cohort 2	Follow-up	iGeneTech ALExome V1-CNV	Illumina NovaSeq	100	95
	Cohort 3	Follow-up	Agilent SureSelect Human All Exon V6	Illumina NovaSeq	30	34
	Cohort 4	Follow-up	Agilent SureSelect Human All Exon V4 or V5 Kit or NimbleGen SeqCap EZ Human Exome V3.0	Illumina HiSeq 2500	200	176
	Cohort 5 (dbGaP)	Follow-up	NimbleGen VCRome 2.1 (HGSC design)	Illumina HiSeq 2500	60	61
Control cohorts	HUABIAO project	Discovery	iGeneTech ALExome V1-CNV	Illumina HiSeq 2500	107	104
	gnomAD (v4.1.0)	Follow-up	Deep whole-genome sequencing or multiple exome capture methods	Multiple sequencing platforms	Not provided	>20
	BRAVO (TOPMed release 10)	Follow-up	Deep whole-genome sequencing	Multiple sequencing platforms	>38	>30
	Regeneron Genetics Center	Follow-up	IDT xGenv1	Illumina NovaSeq 6000	39	>60
	NyuWa Genome resource	Follow-up	Deep whole-genome sequencing	Illumina HiSeq X10 or NovaSeq 6000	>30	Not provided
	ChinaMAP	Follow-up	Deep whole-genome sequencing	BGISEQ-500	>30	Not provided

Supplemental Table 2. ***MGA*** **LoF variants identified in POI patients and their extremely low allele frequencies in human populations.**

MGA variant	Variant type	Affected exon	Allele frequency in populations	
			gnomAD v4.1.0	BRAVO release 10
c.350C>G (p.Ser117*)	nonsense	2	0	0
c.736C>T (p.Gln246*)	nonsense	2	0	0
c.988C>T (p.Arg330*)	nonsense	2	0	0
c.1026_1027insCA (p.Ser343Glnfs*7)	frameshift insertion	2	0	0
c.1173C>A (p.Tyr391*)	nonsense	3	0	0
c.1287del (p.Arg429Serfs*33)	frameshift deletion	3	0	0
c.1673del (p.Asp558Alafs*42)	frameshift deletion	3	0	0
c.1829del (p.Val610Alafs*11)	frameshift deletion	3	0	0
c.2009C>G (p.Ser670*)	nonsense	3	0	0
c.2296dup (p.Thr766Asnfs*9)	frameshift insertion	6	0	0
c.2678_2679del (p.Pro893Argfs*19)	frameshift deletion	8	0	0
c.2686C>T (p.Arg896*)	nonsense	8	0	0
c.2709_2712del (p.Ala905Leufs*27)	frameshift deletion	8	0	0
c.2728C>T (p.Arg910*)	nonsense	8	1.3E-06	0
c.2840C>G (p.Ser947*)	nonsense	8	0	0
c.2914C>T (p.Gln972*)	nonsense	8	0	0
c.3217C>T (p.Arg1073*)	nonsense	9	0	0
c.3463C>T (p.Arg1155*)	nonsense	10	6.6E-06	3.3E-06
c.3661del (p.Arg1221Glyfs*15)	frameshift deletion	11	0	0
c.3739A>T (p.Lys1247*)	nonsense	11	0	0
c.4480del (p.Leu1494Cysfs*25)	frameshift deletion	14	0	0
c.5035_5036insG (p.Leu1679Cysfs*50)	frameshift insertion	15	0	0
c.5062_5063del (p.Leu1688Serfs*40)	frameshift deletion	15	0	0
c.5452C>T (p.Arg1818*)	nonsense	16	1.4E-06	0
c.5504-2A>G (p.Gly1835Glufs*2)	splice site	17	0	0
c.6499_6500del (p.Leu2167Glyfs*27)	frameshift deletion	17	0	0
c.6645dup (p.Gln2216Serfs*8)	frameshift insertion	17	0	0
c.6651_6661del (p.Gln2217Hisfs*3)	frameshift deletion	17	0	0
c.7118del (p.His2373Profs*31)	frameshift deletion	18	0	0
c.7135C>T (p.Gln2379*)	nonsense	18	0	0
c.7139+1G>A (p.Ser2381*)	splice site	18	6.4E-06	0
c.7186C>T (p.Arg2396*)	nonsense	19	6.2E-06	0
c.7396C>T (p.Arg2466*)	nonsense	20	0	0
c.7460_7461dup (p.Leu2488Serfs*2)	frameshift insertion	21	0	0

c.7589_7595del (p.Lys2530Argfs*25)	frameshift deletion	22	0	0
c.7601del (p.Gly2534Aspfs*23)	frameshift deletion	22	0	0
c.7731dup (p.Asp2578Argfs*45)	frameshift insertion	22	6.9E-07	0

Note: The RefSeq accession numbers for *MGA* and its protein are NM_001164273.2 and NP_001157745.1, respectively. NM_001164273.2 has a total of 24 exons.

Supplemental Table 3. Clinical Characteristics in Patients with *MGA* Loss-of-function Variants.

Patient ID	Cohort	Phenotype	Menarche age (yr)	Amenorrhea age (yr)	Diagnosis age (yr)	FSH (U/liter)	LH (U/liter)	E2 (pg/ml)	Reproductive History	CGG repeats in *FMR1* gene (Alleles 1/2)
POI-173	Cohort 1	SA	15	20	NA	127.5	39.0	21.0	G0P0A0	21/30
POI-399	Cohort 1	PA	–	–	–	73.0	15.4	<5.0	G0P0A0	29/29
POI-588	Cohort 1	PA	–	–	–	52.3	20.2	<5.0	G0P0A0	29/29
POI-653	Cohort 1	PA	–	–	–	48.2	15.3	<5.0	G0P0A0	29/29
POI-724	Cohort 1	SA	13	16	16	72.7	83.9	5.0	G0P0A0	30/43
POI-740	Cohort 1	PA	–	–	–	146.4	63.1	<5.0	G0P0A0	30/36
POI-795	Cohort 1	SA	16	33	34	89.0	32.4	NA	G0P0A0	29/29
POI-825	Cohort 1	SA	17	17	NA	80.7	27.8	<5.0	G0P0A0	29/29
POI-830	Cohort 1	SA	19	19	24	75.8	49.1	42.7	G0P0A0	29/29
POI-933	Cohort 1	SA	14	20	20	87.4	42.3	<5.0	G0P0A0	29/29
POI-956	Cohort 1	SA	14	27	27	75.5	63.9	NA	G1P1L1A0	29/30
POI-1060	Cohort 1	SA	16	16	NA	47.8	22.9	<5.0	G0P0A0	30/49
POI-1146	Cohort 1	SA	13	17	17	81.7	39.7	27.0	G0P0A0	30/39
POI-1265	Cohort 1	SA	16	16	16	51.8	34.1	16.6	G0P0A0	30/36
POI-1274	Cohort 1	SA	14	15	25	82.2	49.1	23.2	G0P0A0	29/30
POI-1332	Cohort 1	PA	–	–	–	61.8	35.6	<5.0	G0P0A0	31/36

POI-1385	Cohort 1	SA	13	13	23	48.0	25.4	<5.0	G0P0A0	29/29
POI-1422	Cohort 1	SA	16	17	17	51.0	18.1	46.6	G0P0A0	29/44
POI-1447	Cohort 1	SA	15	24	24	107.0	38.6	29.8	G0P0A0	29/36
POI-1472	Cohort 1	SA	15	20	22	76.3	46.0	18.4	G0P0A0	29/36
POI-1565	Cohort 1	SA	14	NA	NA	38.1	22.6	37.6	G3P0L0A3	29/29
POI-1573	Cohort 1	SA	17	18	18	57.9	39.3	5.0	G0P0A0	29/36
POI-1633	Cohort 1	SA	14	18	18	50.6	32.5	<5.0	G0P0A0	30/40
POI-1712	Cohort 1	SA	15	23	25	137.0	79.5	7.6	G0P0A0	30/36
POI-1729	Cohort 1	SA	16	NA	NA	56.8	33.9	14.0	G0P0A0	29/29
POI-1760	Cohort 1	SA	17	19	22	89.5	42.8	<5.0	NA	29/30
POI-20319	Cohort 1	SA	NA	20	NA	106.4	23.5	38.0	NA	29/29
NP-130	Cohort 2	SA	12	32	32	106.6	65.3	<5.0	G2P1L1A1	30/30
NP-173	Cohort 2	SA	17	26	27	37.0	56.7	<5.0	G0P0A0	36/36
P24545	Cohort 3	SA	14	29	29	47.8	10.9	29.0	G1P1L1A0	29/31
P25024	Cohort 3	SA	14	23	23	125.9	18.2	53.3	G0P0A0	29/32
P25199	Cohort 3	SA	16	20	20	58.3	10.0	23.5	G0P0A0	29/36
P29578	Cohort 3	SA	13	21	21	100.7	NA	NA	G0P0A0	29/30
P30044	Cohort 3	SA	13	21	21	82.4	20.0	34.5	G0P0A0	29/29
P30341	Cohort 3	SA	14	30	30	62.4	40.0	66.6	G0P0A0	28/28

P30429	Cohort 3	SA	13	20	20	53.4	<5.0	22.4	G0P0A0	29/29
IPOF-22	Cohort 4	PA	−	−	−	57.8	72.8	15.0	G0P0A0	This individual has been confirmed to have normal CGG repeats in *FMR1* prior to undergoing exome sequencing without documenting specific repeats number.
POI00796	Cohort 5	NA	NA	NA	NA	NA	NA	NA	NA	Not available

Note: Normal, premutation, and full mutation of CGG repeats in *FMR1* are respectively <55, 55–199, and ≥200.

Abbreviations: −, not applicable; A, abortion; E2, estradiol; FSH, follicle-stimulating hormone; G, gestation; NA, not available; PA, primary amenorrhea; P, production; L, living; LH, luteinizing hormone; SA, secondary amenorrhea.

Supplemental Table 4. Information of female family members with *MGA* LoF variants.

Family	Family member	*MGA* LoF variant	Age of menopause	Reproductive history
Family 3	II-2	c.2709_2712del, p.Ala905Leufs*27	31	G3P1L1A2
Family 3	II-3	c.2709_2712del, p.Ala905Leufs*27	35	G0P0L0A0
Family 4	I-2	c.2728C>T, p.Arg910*	30	G3P1L1A2

Supplemental Table 5. Annotations of upregulated genes in *Mga*$^{+/−}$ mice

Gene	Entrez Gene ID	Description	DEGs in P5	DEGs in 1M	DEGs in 5M
Stag3	50878	STAG3 cohesin complex component	yes	yes	yes
Prr19	623131	proline rich 19	yes	yes	no
Hormad2	75828	HORMA domain containing 2	yes	yes	yes
Spaca1	67652	sperm acrosome associated 1	yes	yes	yes
Zfp541	666528	zinc finger protein 541	yes	no	no
Sycp1	20957	synaptonemal complex protein 1	yes	no	no
Tbr1	21375	T-box brain transcription factor 1	yes	yes	no
8030474K03Rik	382231	RIKEN cDNA 8030474K03 gene	yes	yes	yes
4930447C04Rik	75801	RIKEN cDNA 4930447C04 gene	yes	yes	yes
Gm4779	102634296	predicted gene 4779	yes	yes	yes
Iho1	434438	interactor of HORMAD1 1	yes	yes	yes
Esrrb	26380	estrogen related receptor, beta	yes	no	no
Meiosin	243866	meiosis initiator	yes	yes	yes
Henmt1	66715	HEN1 methyltransferase homolog 1 (Arabidopsis)	yes	no	no
Efcab6	77627	EF-hand calcium binding domain 6	yes	no	no
Rhox5	18617	reproductive homeobox 5	yes	no	no
Taf7l	74469	TATA-box binding protein associated factor 7 like	yes	no	no
Sp9	381373	trans-acting transcription factor 9	yes	no	no
Camk4	12326	calcium/calmodulin-dependent protein kinase IV	yes	no	no
Rad21l	668929	RAD21-like (S. pombe)	yes	no	no
Rbm46	633285	RNA binding motif protein 46	no	yes	yes
Fkbp6	94244	FK506 binding protein 6	no	yes	yes
Zcwpw1	381678	zinc finger, CW type with PWWP domain 1	no	yes	yes
Trank1	320429	tetratricopeptide repeat and ankyrin repeat containing 1	no	yes	no
Gpc2	71951	glypican 2 cerebroglycan	no	yes	no
Rnf17	30054	ring finger protein 17	no	yes	no
Zdbf2	73884	zinc finger, DBF-type containing 2	no	yes	no
Gpat2	215456	glycerol-3-phosphate acyltransferase 2, mitochondrial	no	yes	no
Tchh	99681	trichohyalin	no	yes	no
Rnf157	217340	ring finger protein 157	no	yes	no
Klhl35	72184	kelch-like 35	no	yes	yes
2610037D02Rik	70040	RIKEN cDNA 2610037D02 gene	no	no	yes
Slc25a31	73333	solute carrier family 25 (mitochondrial carrier; adenine nucleotide translocator), member 31	no	no	yes
Zfp783	232785	zinc finger protein 783	no	no	yes

Miat	330166	myocardial infarction associated transcript (non-protein coding)	no	no	yes
Gm15222	102638002	predicted gene 15222	no	no	yes
Xlr4b	27083	X-linked lymphocyte-regulated 4B	no	no	yes

Supplemental Table 6. Frequencies of the individuals with *MGA* missense and inframe variants in POI case cohorts and human genome public databases.

Group	Cohort	Predicted damaging missense	In-frame
Case cohorts	Cohort 1	16 (1.56%)	0 (0%)
	Cohort 2	5 (2.55%)	0 (0%)
	Cohort 3	13 (2.68%)	3 (0.62%)
	Cohort 4	0 (0%)	1 (0.96%)
	Cohort 5 (dbGaP)	2 (2.04%)	0 (0%)
	All cases	36 (1.88%)	4 (0.21%)
Control cohorts	HUABIAO project	49 (0.98%)	3 (0.06%)
	gnomAD (v4.1.0)	7193 (0.89%)	1500 (0.19%)
	BRAVO (TOPMed release 10)	2455 (1.63%)	754 (0.5%)
	Regeneron Genetics Center	1033 (0.75%)	290 (0.21%)
	NyuWa Genome resource	109 (3.63%)	1 (0.03%)
	ChinaMAP	322 (3.04%)	14 (0.13%)
	All controls	11161 (1%)	2562 (0.23%)

Supplemental Table 7. Pathogenic or likely pathogenic variants in other known POI genes co-occurring in patients with *MGA* LoF variants

Case	Gene	Reported inheritance in POI	Genotype	Variant	AF in HuaBiao	AF in gnomAD	CADD
POI-173	*RECQL4*	AR	heterozygous	c.3034T>C (p.Trp1012Arg)	0	0	23.6
POI-588	*ERCC6*	AD	heterozygous	c.2839C>T (p.Arg947*)	0	1.8E-05	41
POI-956	*HFM1*	AR	heterozygous	c.2028_2029del (p.Asp676Glufs*9)	0	0	33
POI-1146	*SPIDR*	AR	heterozygous	c.1181C>G (p.Ser394*)	0	0	32

Abbreviation: AD, autosomal dominant; AF, allele frequency; AR, autosomal recessive.

Supplemental Table 8. Mini-gene primers used in the mini-gene assays.

Primer names	Usage	Sequence (5'-3')
MGA-c.5504wt-F1	Nested PCR	CACTTTGGAAGGCCGAGATG
MGA-c.5504wt-R1	Nested PCR	GGGGTTTGAGACCAGCCTCA
MGA-c.5504wt-F2	Nested PCR	CGTTTGTACAACTCCTGCAC
MGA-c.5504wt-R2	Nested PCR	GGATTACAGGCACCCATCAC
MGA-c.5504wt-KpnI-F	Introduce restriction sites	GCTTGGTACCATGGAAAATGCTGCTCAAATTCCA
MGA-c.5504wt-EcoRI-R	Introduce restriction sites	TGCAGAATTCAGTCTCAAAGAATAAAAGCT
MGA-c.5504-2A>G-F	Introduce the mutation	CTTATTTTTAAACCGGGGTCTGTGATGGGA
MGA-c.5504-2A>G-R	Introduce the mutation	TCCCATCACAGACCCCGGTTTAAAAAATAAG
MGA-c.7139wt-F1	Nested PCR	GAAGCCCCCTTAAAGTGGCA
MGA-c.7139wt-R1	Nested PCR	GCAATCCTCCCACCTCAGGT
MGA-c.7139wt-F2	Nested PCR	CACGGCATTTTTCCTGTTAC
MGA-c.7139wt-R2	Nested PCR	CAACAAGCCCGGCTAATTTG
MGA-c.7139wt-KpnI-F	Introduce restriction sites	GCTTGGTACCATGAATAACTGTGTAGAATACAT
MGA-c.7139wt-EcoRI-R	Introduce restriction sites	TGCAGAATTCAAATATCTTCCTTCTCTACT
MGA-c.7139+1G>A-F	Introduce the mutation	GTCATTCAAACAGCCATAAGTCTTATTTCTC
MGA-c.7139+1G>A-R	Introduce the mutation	GAGAAATAAGACTTATGGCTGTTTGAATGAC
MGA-c.7008wt-F1	Nested PCR	GTGACACATCCCTGTAGTCC
MGA-c.7008wt-R1	Nested PCR	TGAAACCCTGTCTCTACCCA
MGA-c.7008wt-F2	Nested PCR	TCTGGCCTTTTACAGAAAAC
MGA-c.7008wt-R2	Nested PCR	TGGGATTACAGGCACCCATC
MGA-c.7008wt-KpnI-F	Introduce restriction sites	GCTTGGTACCATGGAAAATGCTGCTCAAATTCCA
MGA-c.7008wt-EcoRI-R	Introduce restriction sites	TTTCCTCGAGCCCAGTTTCTGATTAACATC
MGA-c.7008+1G>A-F	Introduce the mutation	GATGATGTAGAAAAGATGGTGAGCCCATTTT
MGA-c.7008+1G>A-R	Introduce the mutation	AAAATGGGCTCACCATCTTTTCTACATCATC
pcMINI-N-F	RT-PCR and sequencing.	CTAGAGAACCCACTGCTTAC
pcMINI-N-R	RT-PCR and sequencing.	GCCCTCTAGACTGGTCATTCCGGCTC

References

1. Li L, Dong J, Yan L, Yong J, Liu X, Hu Y, et al. Single-Cell RNA-Seq Analysis Maps Development of Human Germline Cells and Gonadal Niche Interactions. *Cell Stem Cell.* 2017;20(6):858-73.e4.

2. Zhang Y, Yan Z, Qin Q, Nisenblat V, Chang H-M, Yu Y, et al. Transcriptome Landscape of Human Folliculogenesis Reveals Oocyte and Granulosa Cell Interactions. *Mol Cell.* 2018;72(6):1021-34.e4.

3. Niu W, and Spradling AC. Two distinct pathways of pregranulosa cell differentiation support follicle formation in the mouse ovary. *Proc Natl Acad Sci U S A.* 2020;117(33):20015-26.

4. Zhou Z, Yang X, Pan Y, Shang L, Chen S, Yang J, et al. Temporal transcriptomic landscape of postnatal mouse ovaries reveals dynamic gene signatures associated with ovarian aging. *Hum Mol Genet.* 2021;30(21):1941-54.
